# Glucose tasting depletes intracellular calcium stores and impairs macrophage functionality

**DOI:** 10.1016/j.isci.2025.113770

**Published:** 2025-10-14

**Authors:** Laura Schlautmann, Daniel Burgdorf, Shaunak Ghosh, Alina Schieren, Linda Klümpen, Isabel Stötzel, Julia Bremser, Michael Döngi, Elvira Mass, Valentin Stein, Thomas Quast, Waldemar Kolanus, Thorsten Lang, Eva Kiermaier, Marie-Christine Simon, Sven Burgdorf

**Affiliations:** 1 Life and Medical Sciences (LIMES) Institute, University of Bonn, Bonn, Germany; 2 Institute of Nutrition and Food Sciences, University of Bonn, Bonn, Germany; 3 Institute for Physiology II, University of Bonn, Bonn, Germany; 4 Deutsches Zentrum Immuntherapie, Uniklinikum Erlangen, Erlangen, Germany

**Keywords:** Health sciences, Immunology, Nutrition related to disease, Cell biology

## Abstract

Hyperglycemia or increased blood glucose concentrations is a characteristic and one of the diagnostic criteria for *Diabetes mellitus* and critically contributes to the onset of secondary diseases. We investigated the effect of hyperglycemia on macrophage activation and functionality, demonstrating enhanced priming toward inflammatory cytokines in hyperglycemic macrophages. Additionally, increased glucose concentrations depleted intracellular Ca^2+^ stores by promoting Ca^2+^ release from the ER. We identified taste receptors on macrophages as sensors for hyperglycemia, mediating Ca^2+^ release into the cytosol by activating the IP3 receptor and inhibiting SERCA. Dysregulated Ca^2+^ homeostasis correlated with taste receptor expression and hyperglycemia in both murine and human cohorts. Consequently, glucose-induced Ca^2+^ depletion resulted in altered macrophage functionality, such as ER stress and impaired cell migration. These findings reveal glucose-induced perturbations in Ca^2+^ signaling and homeostasis and demonstrate a regulatory role of taste receptors in macrophages, enhancing our understanding of immune cell dysfunction in diabetes.

## Highlights

• Hyperglycemia disrupts macrophage Ca^2+^ homeostasis via Tas1R3
• Elevated glucose mediates ER Ca^2+^ release via IP3R activation and SERCA inhibition
• Glucose-mediated Ca^2+^ dysregulation impairs migration and induces ER stress
• Hyperglycemia primes macrophages toward proinflammatory cytokines

## Graphical abstract

**Figure:**
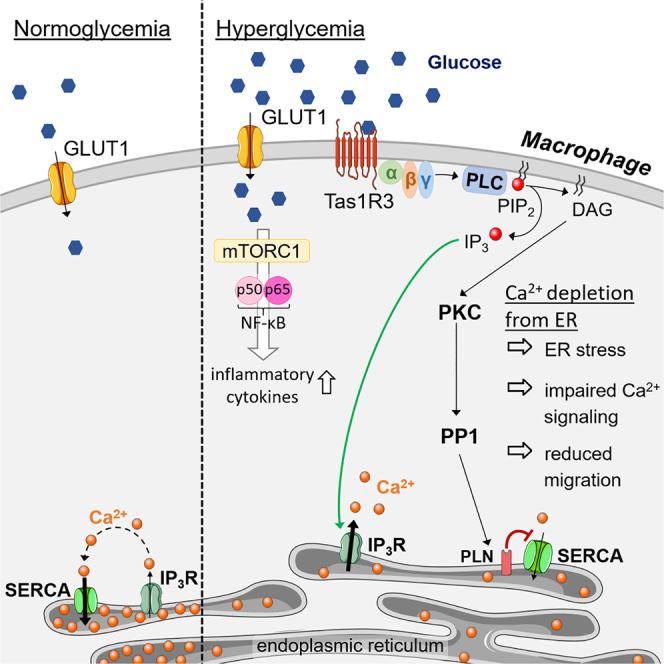

## Introduction

*Diabetes mellitus* is a non-communicable disease, which is characterized by the absence of (type 1 diabetes, T1D) or insensitivity to (type 2 diabetes, T2D) insulin, a major regulator of blood glucose concentrations.^1^ Hence, blood glucose levels of patients with diabetes don't decline efficiently after food intake, leading to hyperglycemia. Fasting blood glucose concentrations are raised from 5.5 mM in healthy people to over 7 mM in patients with diabetes. These chronically elevated blood glucose levels in turn promote diabetes-associated secondary conditions, such as hypertension, heart attacks, strokes, nephropathy, and arteriosclerosis.^2^ The worldwide prevalence of diabetes and diabetes-associated secondary diseases is rising sharply. Accordingly, the identification of the underlying molecular mechanisms has received considerable attention.

Since inflammatory responses induced by hyperglycemia contribute to the onset of diabetes and concomitant secondary diseases, the influence of hyperglycemia on the immune system is of particular interest. In this respect, macrophages play a crucial role, as they can not only induce a strong innate immune response under inflammatory conditions, but also restore tissue repair when inflammation declines.^3^ Therefore, macrophages are considered as gatekeepers at the balance between inflammation and tissue homeostasis.^4^ To fulfill this function, a tight regulation of their signaling is indispensable. Ca^2+^ plays an essential role in the signaling cascades used by macrophages to regulate processes such as differentiation, phagocytosis, and migration.^5^^,^^6^^,^^7^ Under homeostatic conditions, Ca^2+^ is present in high concentrations in the extracellular space (1.1–1.4 mM)^8^ and in intracellular storage compartments such as the endoplasmic reticulum (ER) (1 mM), but in low concentrations in the cytosol (100 nM).^9^ There is constant Ca^2+^ leakage from the ER into the cytosol via a variety of Ca^2+^-permeable channels.^10^ To maintain low cytosolic Ca^2+^ concentrations, the Sarco/endoplasmic reticulum Ca^2+^ ATPase (SERCA) continuously pumps Ca^2+^ into the ER.^11^ Upon macrophage activation, signaling can be amplified by a rapid increase in cytosolic Ca^2+^ concentrations. This initial increase can be induced by Ca^2+^ release from the ER via the activation of the inositol-1,4,5-triphosphate receptor (IP3R), or by Ca^2+^ influx across the cell membrane via Ca^2+^ channels, including P2X receptors and transient receptor potential (TRP) channels.^11^^,^^12^ Importantly, Ca^2+^ efflux across the ER membrane into the cytosol is amplified by influx at the plasma membrane and vice versa, creating a positive feedback loop. Ca^2+^ depletion from the ER translocates STIM near the plasma membrane, where STIM activates the Ca^2+^ channel ORAI. Similarly, the activation of TRP channels at the cell membrane opens IP3R (via phospholipase C (PLC)-mediated generation of inositol-1,4,5-triphosphate (IP3)) and ryanodine receptors (RyRs), both Ca^2+^ channels at the ER.^11^ This results in a rapid and potent cytosolic Ca^2+^ increase and concomitant cellular activation.

In macrophages, excessive glucose can be internalized by glucose transporters (GLUT proteins) for further metabolism.^13^ Alternatively, extracellular glucose can be recognized by the transmembrane sweet taste receptors Tas1R2 and Tas1R3.^14^ It has been reported that sweet taste receptors are generally composed of a heterodimer of Tas1R2 and Tas1R3. However, a homodimer of Tas1R3 or heterodimers with binding partners different from Tas1R2 have also been shown to induce intracellular signaling upon stimulation with glucose.^14^^,^^15^ The sweet taste receptors Tas1R2 and Tas1R3 are G protein-coupled receptors (GPCRs), which, after activation by extracellular glucose or artificial sweeteners, induce the release of the Gβ1γ13 subunit from the associated G protein. The released Gβ1γ13 subunit, in turn, stimulates PLCβ2 to activate protein kinase C (PKC) and to generate IP3,^16^^,^^17^^,^^18^^,^^19^ which finally results in Ca^2+^ release from the ER into the cytosol.^20^

Sweet taste receptors have initially been identified in the taste buds of the tongue. However, various studies report their expression in other organs and cell types,^17^^,^^21^^,^^22^ including macrophages.^23^^,^^24^ Hence, the role of taste receptor function in immune cells has been gaining increasing interest.

In this study, we showed that hyperglycemia results in macrophage activation and perturbates intracellular Ca^2+^ homeostasis. We elucidate how prolonged exposure to enhanced glucose concentrations and concomitant taste receptor activation influences macrophage functionality and the activity of Ca^2+^ channels, Ca^2+^ signaling and Ca^2+^-dependent physiological processes such as cell migration.

## Results

### Elevated glucose concentrations induce pro-inflammatory cytokines via mTOR and NF-κB

**Figure 1:**
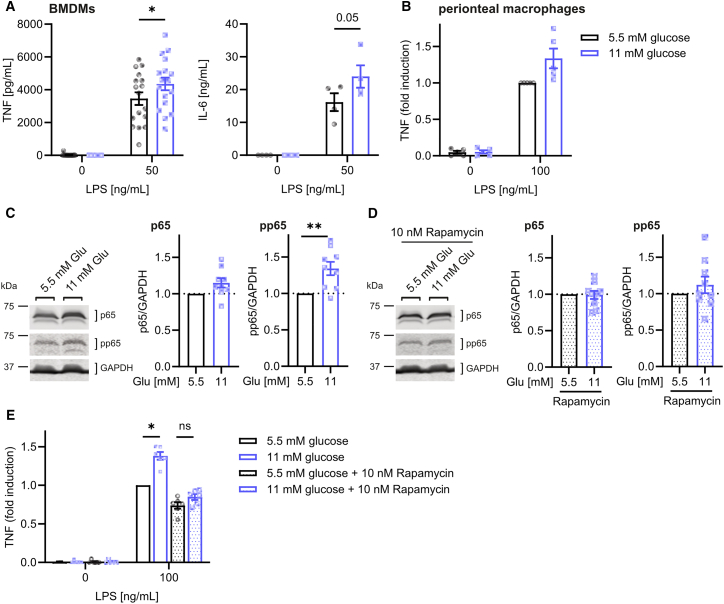
Hyperglycemia induces pro-inflammatory cytokines via mTOR and NF-κB (A and B) Cytokine secretion by LPS-stimulated BMDMs (A) and peritoneal macrophages (B) after treatment with glucose for 48 h, measured by ELISA. (C and D) Phosphorylation of p65 in lysates from BMDMs treated with increased glucose for 24 h in the absence (C) or presence (D) of 10 nM Rapamycin. (E) TNF secretion by LPS-stimulated BMDMs after treatment with glucose and 10 nM Rapamycin for 24 h, measured by ELISA. Data were normalized to levels in untreated BMDMs. Data are presented as mean ± SEM, pooled from independent experiments. ^∗∗^*p* < 0.01, ^∗^*p* < 0.05 by two-way ANOVA corrected for multiple comparisons by the Šidák method (A) or calculation of respective confidence intervals (C–E). Glu: glucose; ns: not significant. See also Figure S1.

To investigate the influence of increased glucose concentrations on macrophage activation, we first analyzed the secretion of pro-inflammatory cytokines in macrophages at hyperglycemia. To this end, cells were kept at physiological concentrations of glucose (5.5 mM), pyruvate (0.1 mM), and glutamine (0.5 mM) 24 h prior to treatment with glucose (final concentrations 11 mM vs. 5.5 mM) for 48 h and stimulation with LPS for another 3 h. We demonstrated that hyperglycemia increased the production of TNF and IL-6 by LPS-stimulated bone marrow-derived macrophages (BMDMs) and peritoneal macrophages (Figures 1A and 1B). The increased production of TNF was prevented by the addition of the GLUT1 inhibitor Bay-876^25^ (Figure S1), suggesting that the observed effects were due to enhanced glucose internalization upon hyperglycemia. Since increased glucose concentrations activate mTOR,^26^ which in turn activates NF-κB,^27^ a major transcription factor driving cytokine production, we investigated whether increased cytokine production was due to the glucose-mediated activation of the mTOR/NF-κB axis. Indeed, we found that increased glucose concentrations enhanced the phosphorylation of p65 (Figure 1C) and observed that increased TNF levels and p65 phosphorylation were both inhibited by the mTOR inhibitor rapamycin (Figures 1D and 1E). These data demonstrate that hyperglycemia enhances macrophage priming for the secretion of inflammatory cytokines via mTOR and NF-κB.

### Hyperglycemia induces the depletion of intracellular Ca^2+^ stores and impairs Ca^2+^ release into the cytosol

**Figure 2:**
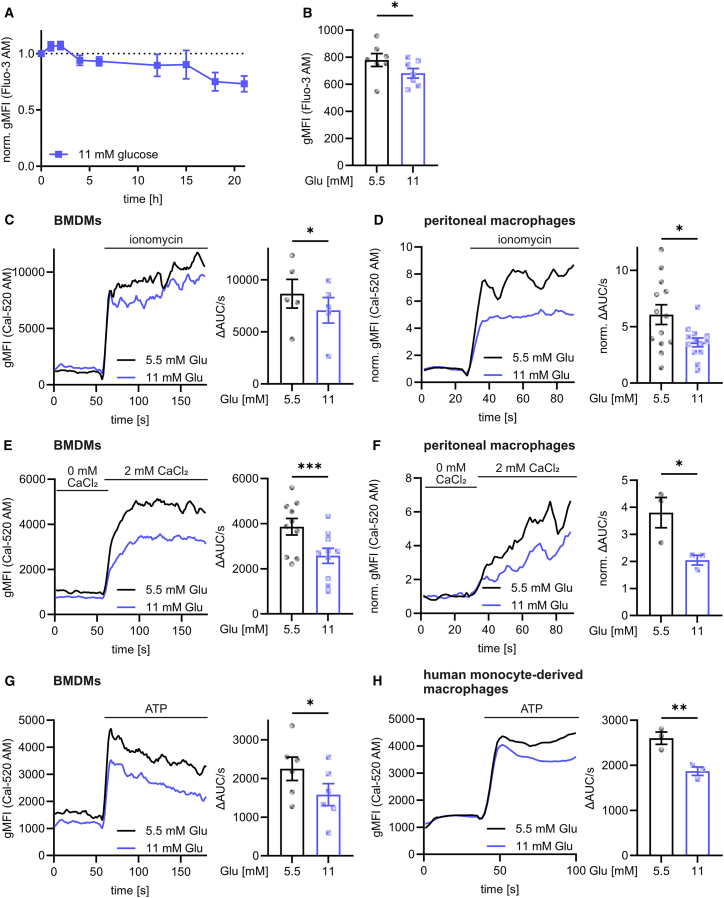
Hyperglycemia induces the depletion of intracellular Ca^2+^ stores and impairs Ca^2+^ release into the cytosol (A) Cellular Ca^2+^ levels after the treatment of BMDMs with 11 mM glucose and staining with Fluo-3 AM by flow cytometry (*n* = 3). (B) As in (A) after 48 h of glucose. (C–H) BMDMs (C, E, and G), peritoneal macrophages (D and F), or human monocyte-derived macrophages (H) were treated with increased glucose for 48 h, stained with Cal-520 AM and Ca^2+^ signaling after addition of 1 μg/mL ionomycin (C and D), 2 mM extracellular CaCl_2_ (E and F) or 100 μM ATP (G and H) was analyzed by flow cytometry. Bar graphs are depicted as mean ± SEM. Ca^2+^ release curves are represented as pooled data from the same set of independent experiments. ^∗∗∗^*p* < 0.001, ^∗∗^*p* < 0.01, ^∗^*p* < 0.05 by paired Student’s t test. ΔAUC/s: difference of the area under the curve per second between after and before stimulation; gMFI: geometric mean fluorescence intensity; Glu: glucose; norm.: normalized. See also Figure S2.

Since Ca^2+^ is an essential regulator of macrophage functionality^5^^,^^28^ and Ca^2+^ homeostasis has been demonstrated to be deranged in patients with diabetes,^29^ we wondered whether enhanced glucose concentrations might disturb intracellular Ca^2+^ homeostasis in hyperglycemic macrophages. To test this hypothesis, we first analyzed the influence of increased glucose concentrations on cellular Ca^2+^ concentrations in macrophages using the Ca^2+^-sensitive dye Fluo-3 AM, which recognized total cellular Ca^2+^ (Figure S2A). Strikingly, after an initial increase, we observed a prolonged decrease in intracellular Ca^2+^ concentrations following the treatment of macrophages with glucose (Figures 2A and 2B). We then investigated whether the glucose-induced depletion of intracellular Ca^2+^ was associated with altered Ca^2+^ signaling and established a flow cytometry-based protocol to monitor changes in cytosolic Ca^2+^ concentrations using the fluorescent cytosolic Ca^2+^ sensor Cal-520 AM (Figure S2A). This technique beared the advantage of measuring time-dependent changes in a large number of cells. We monitored the effect of increased glucose concentrations on Ca^2+^ release into the cytosol after stimulation with the Ca^2+^ ionophore ionomycin, which causes influx of extracellular Ca^2+^ independent of Ca^2+^ channels in the plasma membrane.^30^ Additionally, we induced Ca^2+^ signaling by stimulation with 2 mM extracellular Ca^2+^, which activates metabotropic Ca^2+^-sensitive receptors (CaSRs) at the plasma membrane,^31^ with extracellular ATP, which induces Ca^2+^ signaling via the activation of purinergic receptors,^5^ and with the SERCA inhibitor thapsigargin, which prevents Ca^2+^ transport into the ER and therefore increases cytosolic Ca^2+^ concentrations.^32^ Importantly, all described stimuli induced a reduced Ca^2+^ response in the cytosol of hyperglycemic BMDMs (final glucose concentration 11 mM; Figures 2C, 2E, and 2G; Figures S2B and S2C), murine peritoneal macrophages (Figures 2D and 2F) and human monocyte-derived macrophages (Figure 2H). Impaired Ca^2+^ signaling was also observed in the absence of extracellular Ca^2+^, suggesting that the observed changes might be due to altered Ca^2+^ release from intracellular storage compartments rather than changes in Ca^2+^ influx (Figure S2D). Similar observations were also made using the ratiometric cytosolic Ca^2+^ sensor FuraRed AM (Figure S2E). This showed that the observed differences were not caused by an unequal dye loading efficiency between untreated and glucose-treated cells. In contrast to observed changes in the secretion of pro-inflammatory cytokines, impaired Ca^2+^ translocation into the cytosol was independent of mTOR (Figure S2F) and NF-κB (Figure S2G).

### Glucose modulates Ca^2+^ release into the cytosol from the endoplasmic reticulum

**Figure 3:**
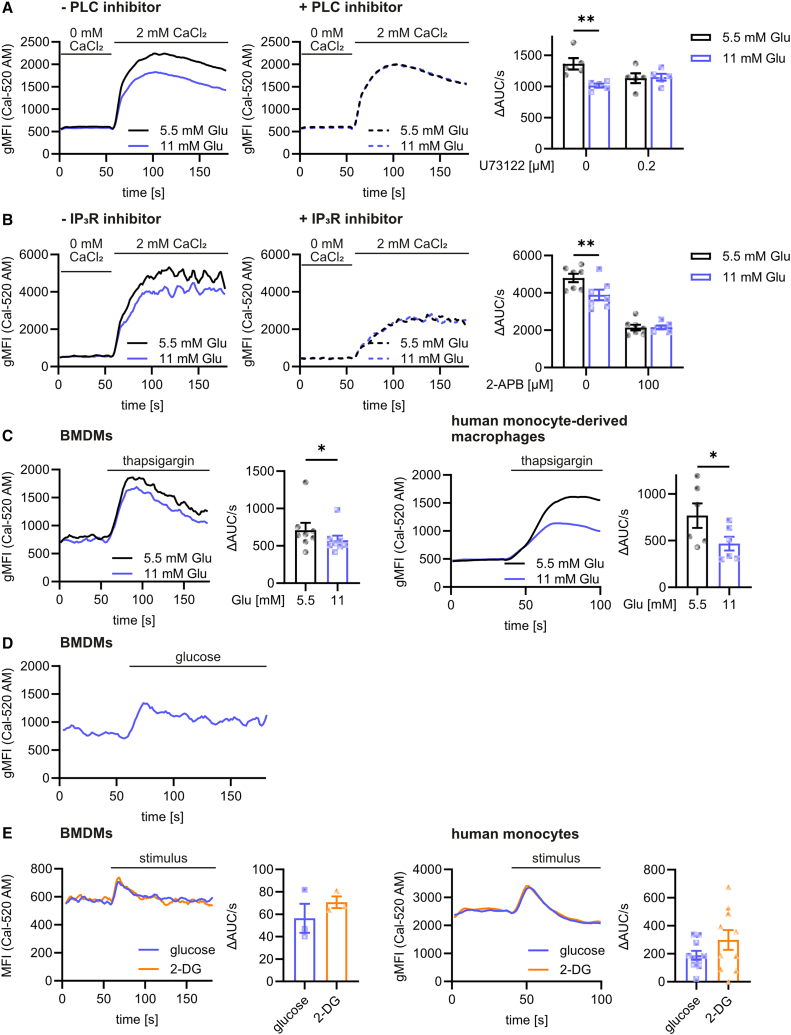
Glucose regulates Ca^2+^ homeostasis at the ER membrane (A) Changes in cytosolic Ca^2+^ concentrations of Cal-520 AM-stained BMDMs pretreated with increased glucose concentrations and the PLC inhibitor U73122 (0.2 μM) for 48 h. Cells were stimulated with 2 mM extracellular CaCl_2_ during analysis. (B) Ca^2+^ signaling of Cal-520 AM-stained BMDMs pretreated with increased glucose concentrations and the IP3 receptor inhibitor 2-APB (100 μM) for 24 h by flow cytometry. (C) BMDMs or human monocyte-derived macrophages were incubated with increased glucose for 48 h, stained with Cal-520 AM and analyzed by flow cytometry in the absence of extracellular Ca^2+^. Cells were stimulated with 1 μM thapsigargin at the indicated time points. (D) BMDMs were stained with Cal-520 AM and analyzed by flow cytometry. Cells were stimulated with 22 mM glucose (*n* = 6) at the indicated time point. (E) As in (D) using BMDMs (left) or human CD14^+^ classical monocytes (right) after stimulation with 22 mM glucose or 2-DG. Bar graphs are depicted as mean ± SEM. Ca^2+^ signaling curves are represented as pooled data from the same set of independent experiments. ^∗∗^*p* < 0.01, ^∗^*p* < 0.05 by two-way ANOVA corrected for multiple comparisons by the Šidák method. ΔAUC/s: difference of the Area under the Curve per second between after and before stimulation; gMFI: geometric Mean Fluorescence Intensity; Glu: glucose; 2-DG: 2-deoxyglucose; norm.: normalized. See also Figure S3.

As described above, there is a substantial correlation between Ca^2+^ transport across the ER and the cell membrane. Ca^2+^ transport across the plasma membrane induces Ca^2+^ release from the ER and vice versa. We therefore intended to investigate whether changes in Ca^2+^ release into the cytosol induced by increased glucose levels were originally due to changes at the cell membrane or rather at the ER. To distinguish between these possibilities, we first induced Ca^2+^ signaling at the cell membrane by the addition of extracellular Ca^2+^ in the presence of the PLC inhibitor U73122 or the IP3R inhibitor 2-aminoethyl diphenylborinate (2-APB). Both inhibitors prevent Ca^2+^ release from the ER after the stimulation of CaSR at the plasma membrane. We demonstrated that reduced Ca^2+^ signaling at high glucose concentrations was overcome by the addition of both inhibitors (Figures 3A and 3B), suggesting that the effect of glucose mainly occurred at the ER. To univocally test this hypothesis, we next induced Ca^2+^ influx into the cytosol by thapsigargin, which induces Ca^2+^ release from the ER, in the absence of extracellular Ca^2+^. Here, the effect of glucose on Ca^2+^ signaling remained unaffected (Figure 3C). Similar observations were obtained using a ratiometric Ca^2+^ sensor (Figure S3A), demonstrating that glucose indeed regulated Ca^2+^ homeostasis at the level of the ER.

Since we observed changes in Ca^2+^ homeostasis at enhanced glucose concentrations, we next analyzed whether glucose itself induces intracellular Ca^2+^ signals. Strikingly, we were able to demonstrate by both flow cytometry and fluorescence microscopy that the addition of glucose immediately induced an increase in cytosolic Ca^2+^ (Figure 3D; Figure S3B), which was not observed after the addition of mannitol (Figure S3C). Then, we investigated whether this immediate Ca^2+^ release into the cytosol was mediated by glucose or rather by glucose-derived metabolites. Interestingly, the addition of pyruvate did not affect the cytosolic Ca^2+^ concentrations (Figure S3C), suggesting that the observed effects might be induced by glucose itself rather than its metabolites. To confirm this hypothesis, we stimulated cells with the glucose analogue 2-deoxy glucose (2-DG), which can not be metabolized any further, and demonstrated that stimulation with 2-DG resulted in a clear Ca^2+^ signal in both murine macrophages and human monocytes (Figure 3E). These data clearly demonstrate that glucose itself induced a Ca^2+^ release from the ER into the cytosol.

### Glucose-induced regulation of Ca^2+^ homeostasis is mediated by taste receptor signaling

**Figure 4:**
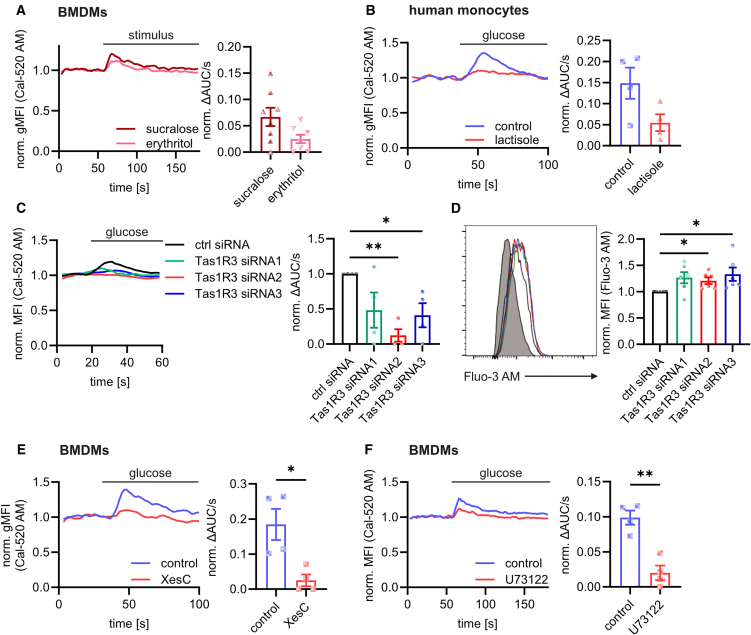
Glucose-induced Ca^2+^ signaling is mediated by taste receptor signaling (A) Changes in cytosolic Ca^2+^ concentrations induced by sucralose (5 mM) and meso-erythritol (5 mM) in BMDMs stained with Cal-520 AM. (B) Glucose-mediated Ca^2+^ signaling in human CD14^+^ monocytes after pretreatment with the Tas1R3 inhibitor lactisole (5 mM). (C) Glucose-mediated (22 mM) Ca^2+^ signaling after siRNA-mediated down-regulation of Tas1R3 in BMDMs. (D) Cellular Ca^2+^ levels after the down-regulation of Tas1R3 measured by Fluo-3 AM using flow cytometry. (E and F) Glucose-induced (22 mM) Ca^2+^ response in BMDMs after pretreatment with 10 μM of the IP3 receptor inhibitor Xestospongin C (E) or 5 μM of the PLC inhibitor U73122 (F). Bar graphs are depicted as mean ± SEM. Ca^2+^ signaling curves are represented as pooled data from the same set of independent experiments. ^∗∗^*p* < 0.01, ^∗^*p* < 0.05 by paired Student’s t test (B, E, and F) or calculation of confidence intervals (C and D). ΔAUC/s: difference of the area under the curve per second between after and before stimulation; ctrl: control; gMFI: geometric mean fluorescence intensity; norm.: normalized; XesC: Xestospongin C. See also Figures S4 and S5.

We next investigated the molecular mechanisms underlying the glucose-mediated regulation of Ca^2+^ homeostasis at the ER membrane. Ca^2+^ homeostasis is generally regulated via G protein-coupled receptors (GPCRs). Since the taste receptors Tas1R2/Tas1R3 are the main GPCR activated by glucose itself,^33^^,^^34^ and Tas1R3 has already been demonstrated to be expressed in macrophages,^23^^,^^24^ we analyzed a putative role of taste receptors in glucose-mediated calcium signaling. To this end, we first stimulated macrophages with 5 mM of sucralose and erythritol, two artificial sweeteners and specific Tas1R3 ligands. Both sucralose and erythritol induced a clear cytosolic Ca^2+^ signal in both murine macrophages and human monocytes (Figure 4A; Figure S4A), suggesting that taste receptors indeed might have been involved. Accordingly, Ca^2+^ signals induced by sucralose, which has been shown to be a very potent Tas1R3 activator,^35^ were more pronounced than those induced by erythritol. In fact, sucralose has been shown to be 600 times more efficient in activating Tas1R3 compared to glucose.^36^ Accordingly, a clear Ca^2+^ release from the ER was still detectable after stimulation with only 0.01 mM of sucralose (Figure S4B), whereas a comparable Ca^2+^ release was induced after addition of 2.75 mM of glucose (Figure S4C). Additionally, glucose-induced Ca^2+^ signaling was inhibited by treating the cells with the human Tas1R3 inhibitor lactisole (Figure 4B), substantiating a decisive role of Tas1R3. Furthermore, the downregulation of Tas1R3 by siRNA (Figure S4D) prevented glucose-induced Ca^2+^ release into the cytosol (depicted by Cal-520 AM) (Figure 4C) and increased overall cellular Ca^2+^ concentrations (depicted by Fluo-3 AM) (Figure 4D), demonstrating that the observed changes in Ca^2+^ homeostasis triggered by increased glucose levels were indeed mediated via Tas1R3. Accordingly, the pretreatment of macrophages with sucralose for 48 h mimicked the glucose-induced reduction of Ca^2+^ release from the ER after the addition of thapsigargin (Figure S4E). Also here, the treatment of macrophages with only very small amounts of sucralose (0.001 mM) still impaired the Ca^2+^ signaling in BMDMs (Figure S4F), highlighting again the potency of sucralose to stimulate Tas1R3 even at low concentrations. Finally, the glucose-mediated impairment of Ca^2+^ release into the cytosol was overcome in Tas1R3-deficient BMDMs (Figure S4G), further substantiating the decisive role of Tas1R3 signaling in the dysregulation of Ca^2+^ homeostasis in hyperglycemic macrophages.

Since the stimulation of Tas1R3 activates PLC, resulting in the generation of IP3, which in turn causes Ca^2+^ release from the ER via the IP3R,^20^ we next investigated whether the glucose-induced stimulation of Tas1R3 caused IP3R-mediated Ca^2+^ release from the ER. Indeed, glucose-induced Ca^2+^ release into the cytosol was diminished after treatment with both IP3R inhibitors Xestospongin C and 2-APB (Figure 4E; Figure S5A). Similarly, glucose-induced Ca^2+^ release was prevented by the PLC inhibitor U73122 (Figure 4F) but not by its inactive analogue U73343 (Figure S5B), demonstrating that the glucose-mediated stimulation of Tas1R3 activated IP3R via PLC. Similarly, the Gβγ inhibitor gallein^37^ prevented glucose-induced Ca^2+^ release (Figure S5C), confirming that the activation of PLC by Tas1R3 was mediated by Gβγ as described previously.^16^^,^^17^^,^^18^^,^^19^ Additionally, hyperglycemia induced increased phosphorylation of IP3R (Figure S5D), which further increases its opening probability.^38^

**Figure 5:**
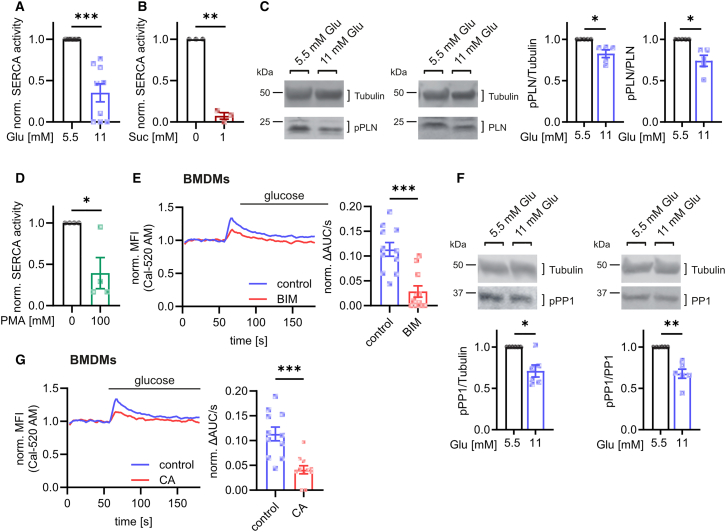
Glucose-mediated activation of Tas1R3 inhibits SERCA via phospholamban dephosphorylation by protein phosphatase 1 (A and B) SERCA activity after 48 h of treatment with glucose (A) or sucralose (B). (C) Western blot analysis after treatment with glucose for 4 h. (D) SERCA activity after stimulation with 100 nM PMA for 24 h. (E) Glucose-mediated Ca^2+^ signaling in Cal-520 AM-stained BMDMs after pretreatment with the protein kinase C inhibitor bisindolylmaleimide (5 μM). (F) Western blot analysis of PP1 phosphorylation after treatment with glucose for 4 h. (G) Glucose-mediated (22 mM) Ca^2+^ signaling in Cal-520 AM-stained BMDMs after pretreatment with the protein phosphatase inhibitor Calyculin A (100 nM). Bar graphs are depicted as mean ± SEM. Ca^2+^ signaling curves are represented as pooled data from the same set of independent experiments. ^∗∗∗^*p* < 0.001, ^∗∗^*p* < 0.01, ^∗^*p* < 0.05 by paired Student’s t test (E and G) or calculation of confidence intervals (A, B, C, D, and F). ΔAUC/s: difference of the Area under the Curve per second between after and before stimulation; BIM: bisindolylmaleimide; CA: calyculin A; Glu: glucose; MFI: mean fluorescence intensity; norm.: normalized; PLN: phospholamban; PP1: protein phosphatase 1; Suc: sucralose. See also Figure S6.

Since IP3R-mediated Ca^2+^ signals are strongly dependent on the Ca^2+^ concentrations in the ER, and these Ca^2+^ concentrations are strictly regulated by SERCA,^39^ we also analyzed SERCA activity at elevated glucose concentrations. We demonstrated that increased glucose and sucralose indeed diminished SERCA activity (Figures 5A and 5B; Figures S6A and S6B), without affecting SERCA expression (Figure S6C). Previous studies have demonstrated that SERCA activity is regulated by inhibition via phospholamban (PLN), which is predominantly expressed in cardiomyocytes,^40^ but moderate amounts can also be found in macrophages (Figure S6D). The inhibitory effect of PLN on SERCA can be overcome by its phosphorylation.^40^ The addition of both glucose and 2-DG induced PLN dephosphorylation (Figure 5C; Figure S6E), suggesting that PLN dephosphorylation indeed might be a key step in SERCA inhibition at high glucose concentrations.

PLN dephosphorylation is regulated by protein phosphatase 1 (PP1),^41^ and the activation of PLC via the induction of DAG stimulates PKC,^20^ an important regulator of PP1.^42^ Therefore, we investigated whether glucose-induced dephosphorylation of PLN and hence the inhibition of SERCA and a resulting Ca^2+^ release from the ER was mediated via PLC/PKC/PP1 signaling. Indeed, the activation of PKC via the addition of phorbol 12-myristate 13-acetate (PMA) blocked SERCA activity to a similar extent as glucose (Figure 5D; Figure S6F). Additionally, blocking PKC using its specific inhibitor bisindolylmaleimide (BIM) decreased glucose-induced Ca^2+^ release from the ER (Figure 5E; Figure S6G), demonstrating that SERCA inhibition and the resulting Ca^2+^ signaling were indeed mediated by the activation of PKC. Moreover, macrophage treatment with elevated glucose concentrations or 2-DG resulted in increased dephosphorylation and hence the activation of PP1 (Figure 5F; Figure S6H), which was prevented by the PKC inhibitor BIM (Figure S6I). Finally, both glucose-induced Ca^2+^ release and PLN dephosphorylation were impaired by the PP1-specific inhibitor calyculin A (CA) (Figure 5G; Figures S6J and S6K).

In addition to PLN, macrophages also express other micropeptides that can bind to and inhibit SERCA activity. From these candidates, another-regulin (ALN) is a more ubiquitously expressed micropeptide,^40^ with substantially higher expression in macrophages compared to PLN (Figure S6L). Notably, the expression of ALN in HEK293T cells impaired intracellular Ca^2+^ release upon stimulation with thapsigargin (Figure S6M), which was diminished even further after the expression of a phosphorylation-deficient (ALN S19A) but not by a phosphomimetic (ALN S19D) ALN mutant^43^ (Figure S6M), suggesting that, similar to PLN, dephosphorylated ALN might also contribute to the inhibition of SERCA at hyperglycemia.

Taken together, these data demonstrate that the glucose-induced activation of Tas1R3 resulted in Ca^2+^ release from the ER and hence in impaired Ca^2+^ signaling by the PLC-mediated activation of IP3R and inhibition of SERCA via PLC/PKC/PP1/PLN signaling.

### Cytosolic Ca^2+^ signaling in macrophages negatively correlates with blood glucose concentrations in murine and human cohorts

**Figure 6:**
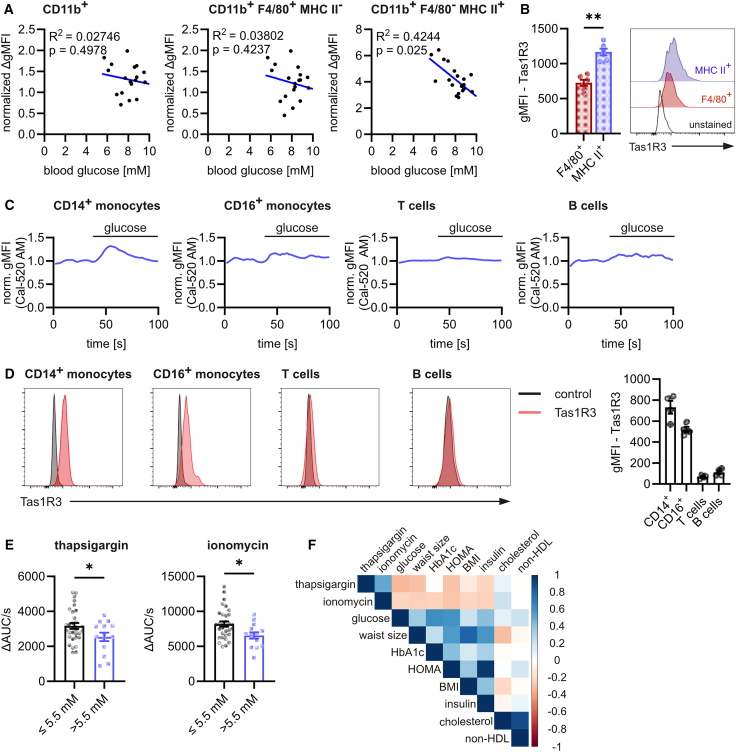
Ca^2+^ signaling negatively correlates with blood sugar concentrations in mice and human cohorts (A) Correlation of CaCl_2_-induced (2 mM) changes in cytosolic Ca^2+^ concentrations with blood sugar concentrations in total (CD11b^+^), large (CD11b^+^ F4/80^+^ MHC II^−^), and small (CD11b^+^ F4/80^-^ MHC II^+^) murine peritoneal macrophages stained with Cal-520 AM. (B) Tas1R3 staining in murine peritoneal macrophages. (C) Glucose-induced (22 mM) changes in cytosolic Ca^2+^ concentrations in human PBMC subsets (*n* = 4). (D) Tas1R3 staining in human PBMC subsets. (E) Ca^2+^ signaling induced by 1 μM thapsigargin (left) or 1 μg/mL ionomycin (right) in CD14^+^ classical monocytes from a human cohort containing 32 healthy (fasting blood glucose ≤5.5 mM) and 14 prediabetic (fasting blood glucose >5.5 mM) donors. (F) Pearson’s correlation of intensity of ionomycin- and thapsigargin-induced Ca^2+^ signaling in CD14^+^ monocytes from (E) to various blood parameters. Data are presented as mean ± SEM. ^∗∗^*p* < 0.01, ^∗^*p* < 0.05 by the calculation of linear regression (A) or paired Student’s t test (B and E). ΔAUC/s: difference of the Area under the Curve per second between after and before stimulation; ΔgMFI: difference in gMFI between after and before stimulation; gMFI: geometric Mean Fluorescence Intensity; norm.: normalized. See also Figures S7 and S8 and Table S1.

Next, we investigated whether the observed correlation between extracellular glucose levels and Ca^2+^ signaling also holds true in a physiological context. Therefore, we first isolated peritoneal macrophages from 9 to 18 months old mice (gated as in Figure S7A) and monitored Ca^2+^ release into the cytosol after stimulation with extracellular Ca^2+^. We observed a weak negative correlation between induced Ca^2+^ signaling and blood glucose concentrations of the mice (Figure 6A). When we distinguished between large (LPM; F4/80^+^ MHC II^−^) and small (SPM; F4/80^-^ MHC II^+^) peritoneal macrophages, we observed a clear negative correlation, especially in SPM (Figure 6A). Interestingly, SPM expressed increased levels of Tas1R3 compared to LPM (Figure 6B), suggesting a putative role of this receptor also in primary macrophages. In line with these observations, we observed a clear reduction in Ca^2+^ signaling in SPM of high fat diet (HFD)-treated mice (Figure S7B), which also depicted increased blood glucose concentrations (Figure S7C).

Additionally, we analyzed Ca^2+^ signals in different subsets of human peripheral blood mononuclear cells (PBMCs) (gated as in Figure S8A). Whereas we observed a clear glucose-induced Ca^2+^ signaling in classical (CD14^+^) monocytes, and moderate Ca^2+^ signaling in non-classical (CD16^+^) monocytes, no glucose-induced Ca^2+^ signaling could be observed in T cells and B cells (Figure 6C). Interestingly, in accordance with the data obtained from murine macrophages, the intensity of Ca^2+^ signaling in human monocytes directly correlated with Tas1R3 expression in these cells (Figure 6D).

Next, we analyzed Ca^2+^ signaling induced by thapsigargin and ionomycin in classical monocytes from a human cohort (*n* = 46) consisting of 32 healthy and 14 prediabetic (fasting blood glucose >5.5 mM) donors (Table S1). As expected, Ca^2+^ signaling intensity after stimulation by thapsigargin correlated with ionomycin-induced Ca^2+^ signaling in the cohort (Figure S8B). Importantly, both thapsigargin- and ionomycin-induced Ca^2+^ release into the cytosol were significantly decreased in classical monocytes from donors with fasting blood glucose concentrations above 5.5 mM (Figure 6E), but not in non-classical monocytes, B cells, and T cells (Figure S8C). These observed differences were independent of donor gender (Figure S8D). In addition, we demonstrated reverse correlations of Ca^2+^ signaling intensity with other glucose-related parameters such as HbA1c, HOMA, blood insulin, BMI, and waist size, but not with total and non-HDL cholesterol (Figure 6F; Figure S8E).

In summary, we demonstrated that increased blood glucose concentrations negatively correlated with Ca^2+^ signaling intensity in Tas1R3-expressing populations of murine macrophages and human monocytes.

### Glucose-induced Ca^2+^ depletion induces endoplasmic reticulum stress and impairs macrophage migration

**Figure 7:**
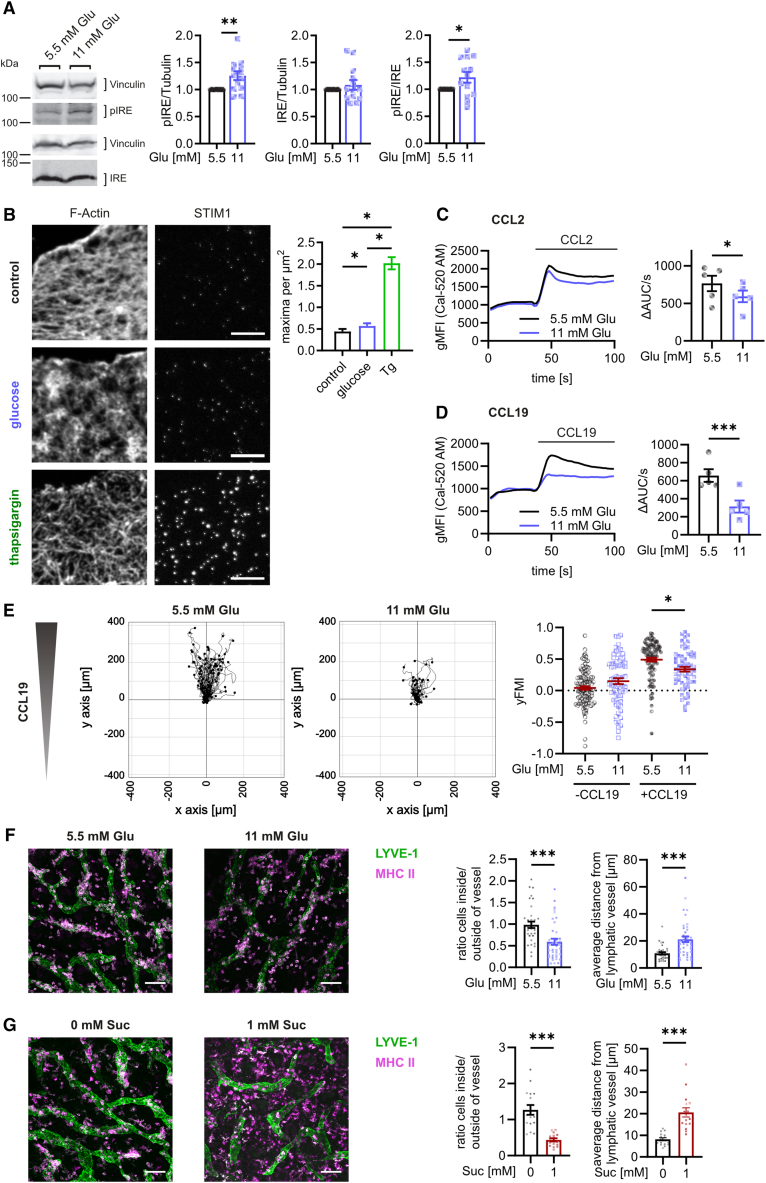
Glucose-induced Ca^2+^ depletion induces ER stress and impairs migration (A) Phosphorylation of IRE1α after the treatment of BMDMs with increased glucose for 48 h by western blot. (B) STIM recruitment toward the cell membrane by STED microscopy. Amount of STIM1 maxima per area was determined in membrane sheets after treatment with additional glucose (final concentration of 11 mM) or thapsigargin (3 μM) for 3 h. Graphs depict pooled data from 3 independent experiments with 15 membrane sheets per sample. Scale bar: 3 μm. (C and D) Changes in cytosolic Ca^2+^ concentrations in response to 14.5 nM CCL2 (C) or 500 ng/mL CCL19 (D) in BMDMs after treatment with glucose for 48 h. (E) BMDMs were treated with increased glucose for 48 h and 200 ng/mL LPS for an additional 18 h. Migration toward 1000 ng/mL CCL19 was analyzed using 3D collagen type I gels. Y-forward migration index (yMFI) was analyzed from time series of live cell imaging. Quantifications depict pooled data from 3 independent experiments with at least 70 cells per condition and region of interest. Migration tracks are shown from one exemplary experiment. (F and G) *In situ* migration in explanted ear sheets incubated with increased glucose (F) or sucralose (G) concentrations for 24 h. Lymphatic vessels were stained for Lyve-1, and migratory cells for MHC II. Data show exemplary Z projections (left) and calculated distance of cells to the lymphatic vessels and ratio of cells in and outside of vessels (right). Each data point represents one field of view pooled from ear sheets from 4 different mice. Scale bar: 100 μm. Bar graphs are depicted as mean ± SEM. Ca^2+^ signaling curves are represented as pooled data from the same set of independent experiments. ^∗∗∗^*p* < 0.001, ^∗∗^*p* < 0.01, ^∗^*p* < 0.05 by calculation of confidence intervals (A), paired Student’s t test (C and D), one-way ANOVA corrected for multiple comparisons by the Tukey method (B and E), and Mann-Whitney test (F and G). ΔAUC/s: difference of the area under the curve per second between after and before stimulation; gMFI: geometric mean fluorescence intensity; Glu: glucose; 2-DG: 2-deoxyglucose; Tg: thapsigargin. See also Figures S9 and S10.

Next, we investigated the functional consequences of glucose-induced dysregulation in Ca^2+^ homeostasis. Ca^2+^ leakage from the ER results in ER stress and STIM translocation toward ER/plasma membrane contact sites.^44^^,^^45^ Additionally, Ca^2+^ depletion from the ER activates the unfolded protein response (UPR) as misfolded proteins accumulate. This generally can be monitored by the phosphorylation of the UPR endonuclease IRE1α, which, upon activation, induces the splicing of XBP1-encoding mRNA.^46^ We demonstrated that treating macrophages with increased glucose concentrations or 2-DG resulted in increased IRE1α phosphorylation and XBP1 splicing (Figure 7A; Figures S9A and S9B), pointing out that increased glucose concentrations indeed induced ER stress. Additionally, we established an approach to visualize and quantify STIM translocation toward ER/plasma membrane contact sites. We used sonication to isolate membrane sheets that remain associated with ER/plasma membrane contact sites as described before.^47^ Afterward, membrane sheets were stained with an STIM-specific antibody, identified by the concomitant staining of the actin cytoskeleton and analyzed by STED microscopy. Using this technique, we demonstrated increased STIM translocation near the plasma membrane after the addition of glucose or 2-DG (Figure 7B; Figure S9C), again confirming the induction of ER stress at hyperglycemia. Accordingly, increased glucose concentrations also decreased surface MHC I expression (Figure S9D), which has been shown to be highly dependent on Ca^2+^ concentrations in the ER and to be reduced at ER stress.^48^

In addition, we investigated functional consequences of glucose-induced Ca^2+^ depletion from the ER. Since macrophage migration depends on Ca^2+^-induced signaling,^7^ we investigated whether Ca^2+^ depletion after glucose treatment also affects cell migration. Indeed, macrophage treatment with the chemokines CCL2 and CCL19 induced a Ca^2+^ release into the cytosol, which was reduced by elevated levels of glucose (Figures 7C and 7D). Similar observations were made after stimulation with sucralose (Figure S10A), again highlighting the importance of Tas1R3. Accordingly, we demonstrated that increased glucose and sucralose concentrations resulted in impaired macrophage migration *in vitro* toward CCL19 as demonstrated using transwell migration experiments (Figures S10B and S10C) and migration assays in 3D collagen (Figure 7E; Figures S10D and S10E). Importantly, the detrimental effect of glucose on cellular migration was overcome by the siRNA-mediated down-regulation of Tas1R3 (Figures S10F and S10G). The addition of different ER stress inhibitors did not overcome the glucose-induced impairment of migration (Figures S10H and S10I), confirming that the perturbation of Ca^2+^ homeostasis and not the induction of the UPR were responsible for the observed effects. Finally, we monitored the influence of increased glucose concentrations on migration in a more physiological setting and analyzed *in situ* migration in explanted ear sheets, which allows quantitative analysis of cellular migration toward lymphatic vessels.^49^ Importantly, migration was severely impaired when ear sheets were incubated in hyperglycemic medium, both after 24 h and 48 h (Figure 7F; Figure S10J), confirming the detrimental effect of high glucose concentrations on cell migration. Again, we observed similar effects after stimulation with sucralose (Figure 7G), confirming the decisive role of Tas1R3 in the glucose-mediated impairment of cellular migration.

## Discussion

In this study, we demonstrated that hyperglycemia primed macrophages for the increased expression of pro-inflammatory cytokines and induced Ca^2+^ release from the ER. This leads to cellular Ca^2+^ depletion and impaired Ca^2+^ signaling. We demonstrated that increased cytokine secretion was initiated by the mTOR-mediated activation of NF-κB after internalization of excessive glucose via GLUT1. In contrast, induction of the release of cellular Ca^2+^ from the ER into the cytosol and hence depletion of cellular Ca^2+^ stores were initiated at the cell membrane via stimulation of taste receptors by extracellular glucose, without the need for glucose internalization. This demonstrates that both pathways are stimulated in parallel and independently from one another.

We used different cohorts to demonstrate the blood glucose-related impairment of Ca^2+^ signaling and monitored reduced Ca^2+^ signaling in monocytes of patients with diabetes. Our data revealed that hyperglycemia directly affects macrophage functionality and intracellular Ca^2+^ homeostasis. Furthermore, we showed that macrophages can taste excessive glucose in the extracellular environment via Tas1R3, which induced the responsible signaling cascades. Consistently, glucose-induced effects on Ca^2+^ homeostasis were only observed in Tas1R3-expressing macrophages and monocytes, but not in (Tas1R3-negative) B and T cells.

We identified that persistent activation of Tas1R3 in hyperglycemic macrophages leads to Ca^2+^ depletion from the ER by both the stimulation of IP3R and inhibition of SERCA. Ca^2+^ depletion from the ER resulted in a compromised release of Ca^2+^ from the ER into the cytosol after stimulation of Ca^2+^-sensing receptors, purinergic receptors, and chemokine receptors, which ultimately resulted in impaired macrophage functionality.

Tas1R3 was originally identified as a sweet taste receptor in the taste buds of the tongue.^33^ More recent studies revealed that Tas1R3 was also expressed and active in other organs.^21^^,^^22^ We extended these findings to the immune system, where Tas1R3-mediated recognition of high glucose concentrations regulates macrophage functionality. Accordingly, Tas1R3-mediated depletion of Ca^2+^ from the ER was also observed after stimulation with sucralose and erythritol, pointing out that the observed effects on macrophage functionality are not merely restricted to hyperglycemia, but might also be induced by enhanced concentrations of artificial sweeteners. These artificial sweeteners are present only in low concentrations in the blood, but are very potent Tas1R3 ligands.^50^^,^^51^ We also demonstrated that artificial sweeteners bind to and activate taste receptors on macrophages, leading to impaired macrophage activity.

In addition, we demonstrated that prolonged elevated concentrations of glucose stimulate taste receptor-induced Ca^2+^ release from the ER via two mechanisms. First, PLC-induced generation of IP3 opens the IP3R, a Ca^2+^ channel in the ER membrane. Second, PLC-induced activation of PKC inhibits SERCA, which transports Ca^2+^ from the cytosol back into the ER. Glucose-induced stimulation of the IP3R was responsible for increased Ca^2+^ release from the ER into the cytosol, causing Ca^2+^ leakage from the ER. Under normal conditions, this Ca^2+^ would be transported back into the ER almost instantaneously by the high activity of SERCA. However, such transport was prevented by glucose-induced, Tas1R3-mediated dephosphorylation of PLN and hence inhibition of SERCA, which prevented the rise of Ca^2+^ levels in the ER again. Typically, SERCA activity is regulated by a family of short ER membrane micropeptides,^52^ which directly bind to SERCA and inhibit its activity. The most prominent SERCA regulator is PLN, which is particularly expressed in cardiomyocytes. In these cells, PLN regulates Ca^2+^-mediated contraction, and its inhibitory effect can be overcome by phosphorylation.^40^ Here, we demonstrated that macrophages also express moderate amounts of PLN and that taste receptor signaling leads to increased PP1-mediated dephosphorylation of PLN and therefore SERCA inhibition. Additionally, other micropeptides, such as ALN, might contribute to the glucose-induced regulation of SERCA in macrophages.

ER-resident chaperones play a crucial role in protein folding in the ER and critically rely on high local Ca^2+^ concentrations. Therefore, hyperglycemia-induced SERCA inhibition and Ca^2+^ depletion from the ER induce the accumulation of misfolded proteins and hence ER stress.^44^ The SERCA inhibitor thapsigargin is most widely used as an experimental inducer of ER stress.^44^ Prolonged high glucose concentrations have been related to altered Ca^2+^ release from the ER^53^ and ER stress before.^54^^,^^55^ Here, we demonstrated that glucose-mediated activation of taste receptors directly induced SERCA inhibition and cellular Ca^2+^ depletion and identified the underlying molecular pathways. In line with our observations, ER stress is increased in classical monocytes of patients with diabetes,^56^ which, as we showed here, express high amounts of Tas1R3.

Hyperglycemia has been demonstrated to dysregulate Ca^2+^ homeostasis throughout the entire body.^57^ In fact, disturbances in Ca^2+^ homeostasis at the ER in skeletal muscles and neurons during diabetes have been shown to contribute to impaired muscle contractility and neuropathies.^58^^,^^59^ It is plausible that glucose-induced depletion of Ca^2+^ from the ER plays a critical role in these processes. Additionally, our data suggest that continuous cellular Ca^2+^ depletion might lead to an ongoing systemic loss of Ca^2+^. Indeed, increased serum Ca^2+^ concentrations have been associated with a greater risk for T2D,^60^^,^^61^^,^^62^ and increased Ca^2+^ concentrations have been detected in the urine of patients with diabetes^29^^,^^63^ and rats.^64^ Interestingly, diabetes was not observed to increase urinary sodium excretion,^64^ suggesting that the specific impairment of Ca^2+^ homeostasis indeed might lead to enhanced Ca^2+^ excretion. Additionally, hypercalciuria was overcome by treatment with insulin,^63^^,^^64^ again highlighting a decisive role of glucose-mediated perturbations in cellular Ca^2+^ homeostasis. In line with these observations, increasing dietary Ca^2+^ intake is inversely related to T2D risk.^65^^,^^66^ Whether this is linked to restored cellular Ca^2+^ levels or enhanced macrophage functionality, however, remains to be investigated.

Previous studies have demonstrated that hyperglycemia induced macrophage dysfunctionality,^67^^,^^68^ which caused impaired wound healing^67^ and rendered patients with diabetes more susceptible to bacterial infections, with 50% enhanced infection rates compared to healthy individuals.^69^^,^^70^ Both T1D and T2D have been associated with poor outcomes in infections. They are associated with enhanced infection severity, hospitalization, and nearly doubled infection-related mortality.^69^^,^^71^^,^^72^ It is plausible that the hyperglycemia-induced impairment of macrophage functionality at least in part contributes to wound healing impairment and to the higher susceptibility to infections in patients with diabetes. Accordingly, after infection with *Klebsiella pneumoniae* or *Mycobacterium tuberculosis*, impaired macrophage migration toward the lung has been monitored in diabetic mice, resulting in increased susceptibility to infection.^73^^,^^74^ It is conceivable that impaired macrophage migration in these studies may be due to perturbations in intracellular Ca^2+^ homeostasis. Similarly, in the context of diabetes and hyperglycemia, Tas1R3 expression is upregulated in various tissues, including the intestine, the lung, and the testes.^75^^,^^76^^,^^77^ Recent studies linked the expression of Tas1R3 to the western diet-induced impairment of the male reproductive system, as well as diabetes-induced exacerbation of lung infection.^76^^,^^77^ Furthermore, the consumption of artificial sweeteners, which are all potent Tas1R3 ligands, is also associated with impaired glucose tolerance, weight gain, metabolic syndrome, and cardiovascular diseases.^78^^,^^79^ This further demonstrates an important role of Tas1R3 in glucose-induced perturbations of immune cells in diabetes and highlights the necessity of continued in depth research to fully understand the role of Tas1R3 and the impact of artificial sweeteners on human health.

Taken together, we have demonstrated that macrophages are able to sense extracellular glucose and artificial sweeteners via Tas1R3, which induces Ca^2+^ depletion from the ER via stimulation of IP3R and inhibition of SERCA. Consequently, hyperglycemia severely affected macrophage functionality, inducing ER stress and impairing Ca^2+^ signaling and Ca^2+^-related cellular functions such as macrophage migration. These data reveal a mechanism by which macrophage function is impaired at hyperglycemia in both mice and humans and advance our understanding of the interplay between metabolic conditions and immune cell function.

### Limitations of the study

This study provides mechanistic insights into the effects of hyperglycemia on macrophage activation and functionality. However, several limitations should be considered.

The experiments were primarily conducted *in vitro* and *ex vivo* using murine and human macrophages or isolated tissues. Although cell culture conditions were chosen to resemble a physiologically relevant environment, such systems cannot fully capture the complexity of *in vivo* responses. The study identified the influence of the taste receptor on Ca^2+^ signaling and altered macrophage functionality due to glucose-induced Ca^2+^ depletion. However, the role of Tas1R3 in shaping macrophage functionality and its contribution to the onset of diabetes and related complications remains elusive. Future studies incorporating *in vivo* models, including cell type-specific conditional taste receptor knockout models, may address these limitations.

Additionally, the observed correlations between blood glucose levels and Ca^2+^ signaling were cross-sectional, limiting causal interpretation.

## Resource availability

### Lead contact

Further information and requests for resources and reagents should be directed to and will be fulfilled by the lead contact, Sven Burgdorf (burgdorf@uni-bonn.de).

### Materials availability

This study did not generate new unique reagents.

### Data and code availability

• All data for this study are within the main/supplementary figures and tables. Primary data and research tools used in this study are available from the lead contact upon request.
• This article did not include original codes.
• Any additional information required to reanalyze the data reported in this article is available from the lead contact upon request.

## Acknowledgments

This work was funded by 10.13039/501100001659Deutsche Forschungsgemeinschaft SFB1454 (Project 432325352) (to S.B., W.K., E.M.) and project 270976260 (to T.L.) and under Germany’s Excellence Strategy EXC2151 (Project 390873048) (to S.B., WK, E.M., E.K.).

## Author contributions

L.S. and S.B. designed research; L.S., D.B., S.G., I.S., and J.B. performed research; T.Q., E.M., W.K., and T.L. contributed new reagents and analytic tools. S.B., T.Q., T.L., and E.K. supervised experiments; L.S., S.B., A.S., L.K., and M.-C.S. performed the human cohort study; S.B., L.S., D.B., S.G., T.Q., T.L., and E.K. analyzed and interpreted data; M.D. and V.S. provided concepts and expertise; S.B. and L.S. wrote the article.

## Declaration of interests

The authors declare no competing interests.

## STAR★Methods

### Key resources table

**Table:** 

REAGENT or RESOURCE	SOURCE	IDENTIFIER
**Antibodies**		

Rat monoclonal anti-mouse IL-6 (clone MP5-20F3)	eBioScience	Cat#14-7061-85; RRID: AB_468423
Rat monoclonal anti-mouse IL-6 (clone MP5-32C11); biotin-conjugated	eBioScience	Cat#13-7062-85; RRID: AB_466911
Rabbit monoclonal anti-NFκB p65 (clone D14E12)	Cell Signaling	Cat#8242; RRID: AB_10859369
Mouse anti-phospho-NFκB pp65 (clone E.949.5)	Invitrogen	Cat#MA5-15181; RRID: AB_10982265
Anti-mouse/rabbit/goat IgG IRDye 680RD/800CW Secondary antibody	Licor	Cat#926-32213; RRID: AB_621848.Cat#926-32210; RRID: AB_621842. Cat#926-32214; RRID: AB_621846. Cat#926-68070; RRID: AB_10956588. Cat#926-68071; RRID: AB_10956166.
Mouse monoclonal anti-GAPDH (clone 6C5)	Sigma-Aldrich	Cat#MAB374; RRID: AB_2107445
Rabbit anti-phospho-IP3R (Ser1756)	Cell Signaling	Cat#3760;RRID: AB_2249436
Rabbit anti-IP3R (clone D53A5)	Cell Signaling	Cat#8568; RRID: AB_1089069
Rabbit anti-Vinculin	Cell Signaling	Cat#4650; RRID: AB_10559207
Mouse monoclonal anti-Vinculin (clone hVIN-1)	Sigma-Aldrich	Cat#V9264; RRID: AB_10603627
Rabbit anti-SERCA2	Cell Signaling	Cat#4388; RRID: AB_2227684
Mouse monoclonal anti-SERCA3 (clone PL/IM430)	Santa Cruz	Cat#sc-81759; RRID: AB_1129372
Goat anti-PLN	Sigma-Aldrich	Cat#SAB2501894
Rabbit anti-phospho-PLN	Cell Signaling	Cat#8496; RRID: AB_10949102
Rabbit anti-phospho-PLN	Sigma-Aldrich	Cat#07-052; RRID: AB_310352
Rabbit anti-phospho-PP1α (Thr320)	Cell Signaling	Cat#2581; RRID: AB_330823
Rabbit anti-PP1α	Cell Signaling	Cat#2582; RRID: AB_330822
Mouse monoclonal anti-Tubulin (clone DM1A)	Cell Signaling	Cat#3873; RRID: AB_1904178
Mouse monoclonal anti-human CD16 (clone 3G8; PerCP/Cyanine5.5-conjugated)	BioLegend	Cat#302028; RRID: AB_893262
Mouse monoclonal anti-human CD3 (clone UCHT1; PE/Cyanine7-conjugated)	BioLegend	Cat#300420; RRID: AB_439781
Mouse monoclonal anti-human CD14 (clone M5E2; APC-conjugated)	BioLegend	Cat#301808; RRID: AB_314190
Mouse monoclonal anti-human CD19 (clone SJ25C1; PE-conjugated)	BioLegend	Cat#363004; RRID: AB_2564126
Rabbit anti-Tas1R3 (AlexaFluor488-conjugated)	Biozol	Cat#BSS-BS-23618R-A488
Rat monoclonal anti-mouse CD45 (clone 30-F11, PerCP/Cyanine5.5-conjugated)	BioLegend	Cat#103132; RRID: AB_893340
Rat monoclonal anti-CD11b (clone M1/70; PE-Cyanine7-conjugated)	Invitrogen	Cat#25-0112-82; RRID: AB_469588
Rat monoclonal anti-F4/80 (clone BM8; eFluor660-conjugated)	Invitrogen	Cat#50-4801-82; RRID: AB_11149361
Rat monoclonal anti-mouse MHC II (I-A/I-E, clone M5/114.15.2; PE-conjugated	Invitrogen	Cat#12-5321-81; RRID: AB_465927
Rat monoclonal anti-mouse CD192 (CCR2; clone SA203G11; APC-conjugated)	BioLegend	Cat#150628; RRID: AB_2810415
Rat monoclonal anti-mouse CD197 (CCR7; clone 4B12; PE-conjugated)	Invitrogen	Cat#12-1971-82; RRID: AB_465905
Mouse monoclonal anti-mouse MHC I (H-2Db; clone 28-14-8)	Invitrogen	Cat#12-5999-81; RRID: AB_466124
Rabbit monoclonal anti-IRE1α (clone 14C10)	Cell Signaling	Cat#3294; RRID: AB_823545
Rabbit anti-phospho-IRE1α (Ser724)	Invitrogen	Cat#PA1-16927; RRID: AB_2262241
Rabbit monoclonal anti-XBP-1s (clone E9V3E)	Cell Signaling	Cat#40435; RRID: AB_2891025
Rabbit monoclonal anti-STIM1 (clone D88E10)	Cell Signaling	Cat#5668; RRID: AB_10828699
Rat monoclonal anti-mouse LYVE1 (clone ALY7)	Invitrogen	Cat#14-0443-82; RRID: AB_1633414
Donkey anti-rat IgG (H+L; AlexaFluor-488-conjugated)	Jackson ImmunoResearch	Cat#715-546-150; RRID: AB_2340849
Rat monoclonal anti-mouse MHC II (I-A/I-E; clone M5/114.15.2; biotin-conjugated)	Invitrogen	Cat#13-5321-85; RRID: AB_466663
Goat anti-rabbit IgG (StarRed-conjugated)	Abberior	Cat#STRED-1002-500UG; RRID: AB_2833015
Mouse monoclonal anti-Flag M2	Sigma Aldrich	Cat#F1804; RRID: AB_262044

**Chemicals, peptides, and recombinant proteins**		

LPS from *Escherichia coli O127:B8*	Sigma-Aldrich	Cat#L4516
Mouse IL-6 recombinant protein, from *Pichia pastoris*	Invitrogen	Cat#RMIL6I
TMB One, ELISA HRP Substrate	Biozol	Cat#KEM-4380A
Rapamycin	Sigma-Aldrich	Cat#553210; CAS: 53123-88-9
Fluo-3 AM	Invitrogen	Caf#F1241
Cal-520 AM	AAT Bioquest	Cat#21130
Adensoin-5’-triphosphate disodium salt (ATP)	Carl Roth	Cat#HN35.2; CAS: 987-65-5
Thapsigargin	Biomol	Cat#10522; CAS: 67526-95-8
Ionomycin calcium salt from *Streptomyces conglobatus*	Sigma-Aldrich	Cat#I0634; CAS: 56092-82-1
Bay 11-7082; NFκB inhibitor	Sigma-Aldrich	Cat#B5556; CAS: 19542-67-7
2-APB; modulator of IP_3_-induced Ca^2+^ release	Sigma-Aldrich	Cat#100065; CAS: 524-95-8
U73122; PLC inhibitor	Cayman Chemical	Cat#70740; CAS: 112648-68-7
Bisindolylmaleimide I; PKC inhibitor	Sigma-Aldrich	Cat#203290; CAS: 133052-90-1
Calyculin A; PP1/PP2A inhibitor	Cayman Chemical	Cat#19246; CAS: 101932-71-2
Zombie Violet Fixable Viability dye	BioLegend	Cat#423113
Lactisol	Cayman Chemical	Cat#18657; CAS: 150436-68-3
Pancoll Human; Cell Separation Medium, Density 1.077 g/mL	PAN Biotech	Cat#P04-601000
Percoll	Sigma Aldrich	Cat#P1644
Recombinant murine MIP-3β (CCL19)	Peprotech	Cat#250-27B
Recombinant murine CCL2 (MCP-1)	Immunotools	Cat#12343383
Transwell polycarbonate membrane cell culture inserts (pore size 0.5 μm)	Corning	Cat#3421
Superscript IV Reverse Transcriptase	Invitrogen	Cat#18090200
Cyanine Cy^3^ Streptavidin	Jackson ImmunoResearch	Cat#016-160-084; RRID: AB_2337244
Phalloidin-iFluor 488 Reagent	Abcam	Cat#Ab176753
Prolong Gold Antifade Mountant	Invitrogen	Cat#P36930
Phorbol 12-myristate 13-acetate (PMA)	Sigma Aldrich	Cat#P8139; CAS; 16561-29-8
Collagen I, Pure Col	Advanced BioMatrix	Cat#5005
Phospho(enol)pyruvic acid monopotassium salt	Sigma Aldrich	Cat#P7127; CAS:4265-07-0
Lactic dehydrogenase, recombinant from *Escherichia coli*, ≥90 U/mg	Sigma Aldrich	Cat#59747
Pyruvate kinase, from *Bacillus stearothermophilus*, 100-300 units/mg	Sigma Aldrich	Cat#P1903
Calcium ionophore A23187 (Calcimycin)	Sigma Aldrich	Cat#C7522; CAS:52665-697
Beta-Nicotinamide-adenine-dinucleotide, reduced disodium salt (NADH)	Biomol	Cat#16132; CAS:606-68-8
Triton X 100	Carl Rot	Cat#3051.2
Bovine Serum Albumin (BSA)	Sigma Aldrich	Cat#9048-46-8
Poly-L-lysine	Sigma Aldirch	P6282
DNA gel stain, Sybr Safe	Invitrogen	Cat#S33102
Lipofectamine 2000 Transfection Reagent	Invitrogen	Cat#11668019
Recombinant human macrophage colony-stimulating factor (M-CSF)	Immunotools	Cat#11343113
4-20% Mini-PROTEAN Precast Protein Gels	Bio-Rad	Cat#4561096
Immunoblot PVDF membrane, 0.2 μm pore size	Bio-Rad	Cat#1620177
Nitrocellulose membrane	GE Healthcare	
Methanol	Sigma-Aldrich	Cat#32213-M
Intercept (TBS) Blocking Buffer	LI-COR Biosciences	Cat#92760003
Fura Red, AM	AAT Bioquest	Cat#21046
Gallein; Gβγ modulator	Sigma-Aldrich	Cat#371708
U73343; inactive analogue of U73312	Sigma-Aldrich	Cat#662041
Xestospongin C; modulator of IP_3_-induced Ca^2+^ release	Sigma-Aldrich	Cat#2628
Bay-876; GLUT1 inhibitor	Sigma-Aldrich	Cat#SML1774
Liraglutide	Sigma-Aldrich	Cat#SML3925
Morin Hydrate	Sigma-Aldrich	Cat#M4008
Quercetin	Sigma-Aldrich	Cat#PHR1488

**Critical commercial assays**		

Mouse TNF-alpha DuoSet ELISA	R&D Systems	Cat#DY410
Quick-RNA Miniprep Kit	Zymo Research	Cat#R1054

**Experimental models: Cell lines**		

Mouse: J558L GM-CSF producing line	Lutz et al.^80^, Burgdorf et al.^81^	N/A

**Experimental models: Organisms/strains**		

Mouse: C57BL/6	Own breeding	N/A
Mouse: C57BL/6J Tas1R3^-/-^ (Damak et al.^34^)	Bone marrow was kindly provided by H. Wang, Monell Chemical Senses Center Philadelphia	N/A

**Oligonucleotides**		

Stealth siRNA targeting sequence - murine Tas1R3 #1	Thermo Fisher	Cat#MSS234755
Stealth siRNA targeting sequence - murine Tas1R3 #2	Thermo Fisher	Cat#MSS234756
Stealth siRNA targeting sequence - murine Tas1R3 #3	Thermo Fisher	Cat#MSS234757
AllStars Negative control siRNA	Qiagen	Cat#1027281
Primer: XBP-1 Forward – GGTCTGCTGAGTCCGCAGCA	This paper, adapted from Yoon et al.^82^	N/A
Primer: XBP-1 Reverse – AAGGGAGGCTGGTAAGGAAC	This paper, adapted from Yoon et al.^82^	N/A
Primer: GAPDH Forward – ACCATCTTCCAGGAGCGAGA	This paper, adapted from Yoon et al.^82^	N/A
Primer: GAPDH Reverse – GGGCCATCCACAGTCTTCTG	This paper, adapted from Yoon et al.^82^	N/A
Primer: Oligo(dT) -AAAAAAAA AAAAAAAAAA	This paper	N/A
Primer: PLN Forward BamHI -GCGCG CGGATCCATGGAAAAAGTGCAATAC CTCACTCG	This paper	N/A
Primer: PLN Reverse XhoI -GATCGA CTCGAGTCACAGAAGCATCACAAT GATGCAG	This paper	N/A
Primer: ALN Forward BamHI – GCGC GCGGATCCATGGAGGTGAGCCAG GCGGCGTCTGG	This paper	N/A
Primer: ALN Reverse XhoI – GATCGA CTCGAGTCAGGGCAAGAGGTACAC GAAGACG	This paper	N/A
Primer: Beta Actin Forward – CTAAGGCCAACCGTGAAAAG	This paper	N/A
Primer: Beta Actin Reverse - ACCAGAGGCATACAGGGACA	This paper	N/A

**Recombinant DNA**		

Plasmid: pCMV-Tag2B	Stratagene	N/A

**Software and algorithms**		

ImageJ	Schneider et al.^83^	https://imagej.nih.gov/ij/
Manual Tracking plug-in in Image J	National Institute of Health	https://imagej.net/ij/plugins/track/track.html
Chemotaxis and Migration plug-in in ImageJ	Ibidi	N/A
FlowJo	BD Biosciences	Version 10image j
Prism, Version 10	GraphPad Software	N/A
Excel/Word, Office Standard 2019	Microsoft Office	N/A
Affinity Designer	Serif	N/A
R, Version 4.3.1	R foundation	https://www.R-project.org/

**Other**		

BD LSR II, Symphony A5	BD Biosciences	N/A
Microplate reader Infinite 200 PRO	Tecan Trading AG	N/A
Miscroscopes: Olympus IX81, Nikon TI2, Nikon Eclipse TE2000, Zeiss LSM 880 Airyscan, Zeiss Axio	Olympus, Nikon, Zeiss	N/A
Gene Pulser XCell Electroporation Systems	BioRad	N/A
4-channel STED microscope	Abberior Schmidt et al.^84^	N/A
Sonoplus GM2070 sonifier	Bandelin	N/A

### Experimental model and study participant details

#### Mice

If not indicated differently, mice without genetic modifications (C57BL/6) at an age of 8-12 weeks were used. Mice were held under specific pathogen-free conditions at the institutional animal facility and both sexes were used. Mice were sacrificed by cervical dislocation or CO_2_ euthanization.

All investigations have been in accordance with the guidelines from Directive 2010/63/EU of the European parliament and were approved from the local institutional animal care committee (Landesamt für Natur, Umwelt und Verbraucherschutz (LANUV), North Rhine Westphalia).

To generate murine bone marrow-derived macrophages, both male and female mice were used and results from individual experiments were pooled.

To analyze the role of Tas1R3, bones from C57BL/6J Tas1R3^-/-^ mice were kindly provided by H. Wang, Monell Chemical Senses Center, Philadelphia.

To analyze the impact of long-term obesity, female 5-6-week-old mice were put on a control diet (CD, sniff E15748-047) or high-fat diet (HFD, sniff E15742-347) (Az 81-02.04.2019.A146).^85^ At an age of 50-60 weeks, mice were euthanized with CO_2_. For analysis of peritoneal macrophages from aging mice, both male and female mice between 48 and 65 weeks were used.

Blood glucose concentrations were determined using an Accu-Chek Instant blood sugar measuring device in a blood drop taken from the tail.

#### Cell lines

Murine J558L cells were exclusively used as a source for GM-CSF, which was harvested from the cell supernatant, quantified by ELISA and used for the generation of bone marrow-derived macrophages. Supernatant was tested negatively for the presence of mycoplasms before its use in experiments.

#### Human samples

A cohort of 46 Caucasian participants was recruited at the University of Bonn. Participants with fasting blood glucose > 5.5 mM were considered prediabetic. Similar results were obtained for both genders as described in the study. This study complied with all relevant ethical regulations and was conducted in accordance with the principles of the Declaration of Helsinki and its subsequent amendments, approved by the ethics committee of the Medical Faculty, University of Bonn. Written informed consent was obtained from all the participants. Fasting venous blood samples were collected in the morning between 8:00 a.m. and 10:00 a.m. after a 12h overnight fast. Blood was used for isolation of PBMCs as well as clinical characterization (as shown in Table S1). Additional anthropometric measurements (weight, waist size, blood pressure) were determined in the study center.

For all other analyses, blood was taken from healthy donors after written consent and approvement by the ethics council of the University Hospital of Bonn (approval 009/21).

### Method details

#### Isolation of human PBMCs and monocytes

Human PBMCs were isolated from buffy coats or fresh blood collected in EDTA-containing cuvettes from healthy and prediabetic donors. After density gradient centrifugation with Pancoll (density 1.077 g/mL), cells were centrifuged at low speed (200 x g) to remove platelets. PBMCs were either directly used for experiments or further processed for monocyte isolation. To this end, the monocyte fraction was isolated using a hyper-osmotic density gradient centrifugation (48.5% Percoll in 0.16 M sodium chloride solution). Human macrophages were generated from monocytes by differentiation in 100 μg/mL Macrophage Colony-Stimulating Factor (M-CSF). Medium was exchanged after 3 days of culture to a medium containing physiological amounts of glucose (5.5 mM), glutamine (0.5 mM) and pyruvate (0.1 mM) for another 3 days.

#### Isolation and culture of peritoneal macrophages

Peritoneal cells were isolated by intraperitoneal injection of 2 mM EDTA in PBS. Isolated cells were either directly used for experiments or cultured in medium with either physiological (5.5 mM) or elevated (11 mM) glucose concentrations for 48h.

#### Generation of murine BMDMs

BMDMs were generated as described before.^86^ Briefly, bone marrow progenitor cells were differentiated in IMDM (5.5 mM glucose) containing 2.5% supernatant of the Granulocyte Macrophage Colony-Stimulating Factor (GM-CSF)-producing cell line J558 (total GM-CSF concentration: 25 ng/ml). After 6 days, cells were incubated in DMEM containing physiological concentrations of glutamine (0.5 mM), glucose (5.5 mM) and sodium pyruvate (0.1 mM). After another 24h, additional glucose, sucralose, 2-DG and various inhibitors were added for indicated time periods.

#### Cytokine secretion assays

Cells were stimulated with LPS for 3h. Levels of secreted cytokines (TNF and IL-6) into the supernatant were determined by enzyme-linked immunosorbent assay (ELISA).

#### Cell lysis and western blot analysis

For whole cell lysates, cells were lysed in 50 mM Tris-HCl (pH 7.5), 1 mM EGTA (pH 8.0), 1 mM EDTA (pH 8.0), 10 mM β-glycerolphosphate, 50 mM sodium fluoride, 5 mM sodium pyrophosphate, 1 mM sodium vanadate, 0.27 M sucrose, 1% Triton-X by freezing at -20°C. After removal of cellular debris, proteins were separated by size in a polyacrylamide gel and transferred to nitrocellulose membranes by a wet electroblotting in transfer buffer (25 mM Tris, 192 mM Glycin). For analysis of Phospholamban (PLN), proteins were separated using a 4-20% Gradient Gel and transferred onto PVDF membranes (0.2 μm pore size) in transfer buffer with additional 20% methanol. Blocking (1h, room temperate) and antibody stainings were performed in Intercept Blocking Buffer.

#### Monitoring Ca^2+^ levels and Ca^2+^ signaling by flow cytometry

To analyze intracellular Ca^2+^ levels, cells were stained with Cal-520 AM (5 μM), Fluo-3 AM (1 μg/mL) or FuraRed AM (5 μM) for 30 min in HBSS. Fluo-3 AM was used to monitor overall cellular Ca^2+^ levels and the cytosolic sensors Cal-520 AM and FuraRed AM to monitor cytosolic Ca^2+^. For stimulation assays, changes in cytosolic Ca^2+^ concentrations in response to addition of various stimuli and if required after pretreatment with inhibitors for 10 min was monitored over time by flow cytometry. Typically, at least 12.000 cells (300 events/s) were recorded before cells were stimulated. After stimulation, the measurement was continued for at least another 18.000 cells (300 events/s). All changes in intracellular Ca^2+^ concentration analyses were generally depicted as pooled data from independent experiments. All flow cytometric analyses were performed using a FACS LSR II or Symphony A5 (BD Biosciences) and analyzed with the FlowJo Software. FuraRed signals were analyzed by calculation of the ratio of the emission from the Violet laser (excitation: 405 nm, emission: 677 ± 10 nm) over emission from the Yellow-Green laser (excitation: 561 nm, emission: 670 ± 15 nm).

For analysis, the kinetics platform in FlowJo was used for the gated population of interest, which enables the analysis of fluorescence intensity in dependency of the time and the division of the data into time ranges. From this, a time series with values of geometric Mean Fluorescence Intensity (gMFI) for every second of the measurement were exported. Time-resolved gMFI values were then used for graphical representation as well as quantitative analysis. Intensities of changes in cytosolic Ca^2+^ concentrations were calculated from the difference (Δ) of the Area Under the Curve (AUC) for each time frame normalized to one second between measurement before and after stimulus or via differences in the gMFI. For normalization of Ca^2+^ curves, data were normalized to the mean of the baseline.

#### Microscopic analysis of Ca^2+^ release into the cytosol

BMDMs were plated on cover slips and stained with Cal-520 AM as described above. Live cell imaging (one image per second) was performed after addition of ionomycin (1 μg/mL) or glucose (22 mM). After stimulation with ionomycin, images were acquired using a TS100 microscope (Nikon) at a 60x magnification. Fluorescence Intensity of four randomly chosen cells per image was determined and normalized to the baseline after background subtraction. After stimulation with glucose, images were acquired at a TI2 microscope (Nikon) at a 40x magnification. Image analysis was performed using ImageJ.

#### siRNA-mediated downregulation of Tas1R3

After six days of culture, BMDMs were electroporated with 5 μg of control or Tas1R3-specific siRNA using a Gene Pulser XCell Electroporator (Bio-Rad). Two square pulses of 1000 V for 0.5 ms were applied with a time interval of 5 s. Cells were plated and medium was changed to physiological concentrations of glutamine (0.5 mM), glucose (5.5 mM) and sodium pyruvate (0.1 mM) 4h after electroporation. Cells were analyzed after another 2 days.

#### SERCA activity assay

SERCA activity was determined using an indirect enzyme-linked spectrophotometric assay as described before.^87^ BMDMs treated with 11 mM glucose (48h), 5 mM sucralose (48h) or 100 nM PMA (24h) were homogenized in 250 mM sucrose, 5 mM HEPES (pH 7.0) with protease inhibitor. Homogenates (final protein concentration 30 μg/mL) were 20-fold diluted in reaction buffer (100 mM potassium chloride, 10 mM magnesium chloride, 20 mM HEPES, 10 mM phosphoenolpyruvate, 1 mM EGTA, 0.5 mM NADH, 2 μM calcimycin, 15 U/mL pyruvate kinase, 15 U/mL lactic dehydrogenase and 5 μM calcium chloride). Optical density (OD) was determined at 340 nm. After baseline measurement, decrease in OD equivalent to NADH oxidation was monitored over time at 37°C after addition of 5 mM ATP. Specific SERCA activity was determined as the difference between total activity and activity in the presence of the SERCA inhibitor thapsigargin (10 μM).

#### Migration assays

Migration assays were performed as described before.^49^^,^^88^^,^^89^

For *in vitro* migration assays, after incubation with elevated glucose (11 mM) or sucralose (1 mM) concentrations for 48h, BMDMs were stimulated with 200 ng/mL LPS overnight. Transwell migration assays were performed using membrane inserts with a pore size of 5 μm and after addition of 200 ng/mL CCL19 to the lower compartment. Chemotactic migration was assessed after 3h. For 3D migration assays in collagen, BMDMs were mixed with ice-cold collagen solution (Collagen I with 0.7% Sodium Bicarbonate and 1x MEM) and filled into custom-made chemotaxis chambers. After collagen polymerization at 37°C and addition of 1000 ng/mL CCL19 in medium on top of the gel, migration of cells was monitored by automated microscopy every 5 min for 3h at 37°C using a TE Eclipse microscope (Nikon) with a 10x phase-contrast objective. For every experiment, at least 70 individual cells per condition and region of interest were tracked using ImageJ. Calculation of forward migration index (FMI), velocity and accumulated distance as well as visualization of migratory paths was performed using the chemotaxis and migration tools of ImageJ. The FMI is analogous to a chemotactic index and used to compare a cell’s most direct path to the chemokine gradient source to its total accumulated distance.

*For in situ* migration in explanted ear sheets, ventral ear sheets from female 5-week-old mice were placed upside down into medium containing 5.5 or 11 mM of glucose and incubated at 37°C with 5% CO_2_ for 24h or 48h. Ear sheets were fixed in 4% paraformaldehyde (PFA) overnight and permeabilized in 0.2% Triton in PBS for 20 min. After blocking in 1% BSA for 1h, primary antibodies against LYVE-1 (1:200) and MHC II (1:400) were incubated overnight at room temperature, followed by incubation with secondary antibodies (1:400) for 1h. Between every step, ear sheets were washed 3 times in PBS for 10 min. Samples were mounted on microscopy slides with the ventral side towards a protecting cover slip and analyzed by confocal microscopy using an LSM700 microscope (Zeiss). The distance between cells and lymphatic vessels was quantified using Matlab as described before.^49^

#### STED microscopic analysis of STIM1 aggregates

To analyze STIM1 aggregation near the plasma membrane, membrane sheets were analyzed by STED microscopy. For this purpose, BMDMs were plated onto poly-L-Lysine-coated coverslips and treated with thapsigargin (3 μM), glucose (5.5 mM) or 2-DG (5.5 mM) for 3h. After washing with PBS, cover slips were placed into ice-cold sonication buffer (120 mM potassium glutamates, 20 mM potassium acetate, 10 mM EGTA, 20 mM HEPES, pH7.2) and membrane sheets were generated by sonication for 100 ms. Membrane sheets were fixed for 20 min in 4% PFA in PBS, followed by quenching for 20 min with 50 mM ammonium chloride in PBS. After permeabilization in 0.2% Triton in PBS for 1 min, washing with PBS and blocking with 3% BSA for 1h, sheets were stained with anti-STIM1 for 1h (1:200 in blocking solution) and with a Star Red-labeled secondary antibody (1:200 in blocking solution) and iFluor488-conjugated Phalloidin (1:1000) for another 1h at room temperature. Afterwards, membrane sheets were washed three times with PBS. Cover slips were mounted in mounting medium (ProLong Gold antifade reagent) and sealed with nail polish.

Samples were analyzed by confocal and STED microscopy employing a 4-channel STED microscope from Abberior Instruments (available at the LIMES institute imaging facility, Bonn, Germany). The data acquisition was performed as described before.^84^ Per biological replicate, 15 membrane sheets were imaged and preprocessed by introducing a Gaussian Blur (σ = 0.5). All images were analyzed using a custom-made macro for Fiji ImageJ,^84^ which was applied on regions of interest and counted the maxima using a minimum noise tolerance of 5 (a.u.). Samples were simultaneously stained against STIM and ORAI. All images were digitally corrected for cross-excitation as described before.^84^ Each region of interest was selected based on the according Phalloidin staining reference and measured for size to calculate maxima per μm^2^.

#### Semi-quantitative analysis of XBP1 splicing and expression of PLN and ALN via RT-PCR and qPCR

Cells were incubated with 2-DG (5.5 mM) or thapsigargin (500 nM) for 4h and lysed in 300 μL RNA Lysis Buffer (Zymo Research). RNA was isolated using the Quick-RNA Miniprep Kit (Zymo Research) according to manufacturer’s instructions. RNA (5 μg) was reverse transcribed to cDNA with the SuperScript IV Reverse Transcriptase. cDNA was amplified by PCR reaction using primers for XBP1,^82^ GAPDH,^82^ Actin, ALN or PLN. Expected sizes were 256 bp (XBP1), 230 bp (spliced XBP1), 350 bp (GAPDH), 222 bp (ALN) or 183 bp (PLN). Quantitative PCR (qPCR) was performed using the ABsolute SYBR Green Mix (Thermo Fisher) according to Manufacturer’s Instructions. The relative expression levels of PLN and ALN were calculated using the ΔCt method with beta-acting as the reference gene.

#### Cloning of PLN into pCMV-Tag2B and transfection of HEK293T cells

Murine PLN was amplified from cDNA of BMDMs using primers introducing BamHI and XhoI cutting sites and then cloned into a pCMV-Tag2B plasmid. Plasmids were transfected into HEK293T cells using Lipofectamine .

### Quantification and statistical analyses

Statistical analyses were performed using GraphPad Prism, R or Excel. All data were depicted as mean ± standard error of the mean (SEM). The following statistical tests were conducted as noted in the respective figure legend: For comparison between two groups, either paired or unpaired Student’s t test were applied depending on whether the samples were matched or independent. For unmatched data with two groups that do not follow the assumption of normality or equal variance, the Mann-Whitney test was used. One-Way ANOVA followed by a post hoc test was used to compare more than two groups. Depending on whether the means were compared to a control mean or every mean were compared with every mean, data were corrected for multiple comparisons by the Dunnett or the Tukey method, respectively. Two-Way ANOVA was applied to analyze datasets with two independent variables. For post-hoc correction for multiple comparison following Two-Way ANOVA, the Šidák method was used for selected or independent comparisons, whereas the Tukey method was applied when comparing all group means with each other.

For normalized data, confidence intervals (95%, 99% and 99.9%) were calculated and values were considered significant if the interval did not include 1. Correlation was determined by simple linear regression to assess linear relationships or calculation of Spearman correlation to calculate the relation for non-linear associations. Statistical significance is indicated at ∗*p* < 0.05, ∗∗*p* < 0.01, ∗∗∗*p* < 0.001.
