# Supplementary material for: Glucose tasting depletes intracellular calcium stores and impairs macrophage functionality
Source: iScience. 2025 Oct 14;28(11):113770. doi: 10.1016/j.isci.2025.113770 (PMC12616019; doi:10.1016/j.isci.2025.113770)
Supplement: Document S1. Figures S1–S10 and Table S1 [file mmc1.pdf]

iScience, Volume *28*

Supplemental information

Glucose tasting depletes intracellular calcium stores and impairs macrophage functionality

Laura Schlautmann, Daniel Burgdorf, Shaunak Ghosh, Alina Schieren, Linda Klümpen, Isabel Stötzel, Julia Bremser, Michael Döngi, Elvira Mass, Valentin Stein, Thomas Quast, Waldemar Kolanus, Thorsten Lang, Eva Kiermaier, Marie-Christine Simon, and Sven Burgdorf

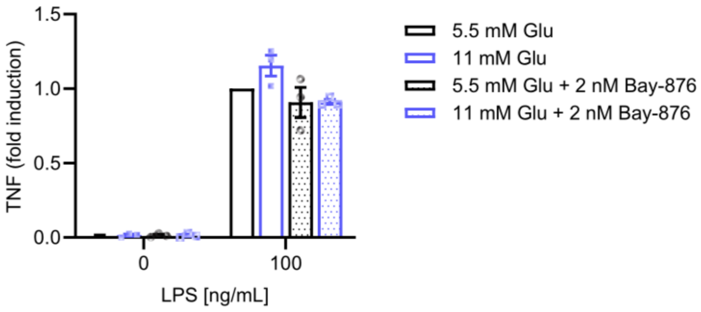

Figure S1, related to Figure 1. The GLUT1 inhibitor Bay-876 overcomes glucose-induced stimulation of cytokine secretion

TNF secretion by LPS-stimulated BMDMs after treatment with glucose and 2 nM Bay-876 for 24h, measured by ELISA. Data were normalized to levels in untreated BMDMs.

Data are presented as mean ± SEM pooled from independent experiments. Glu: glucose.

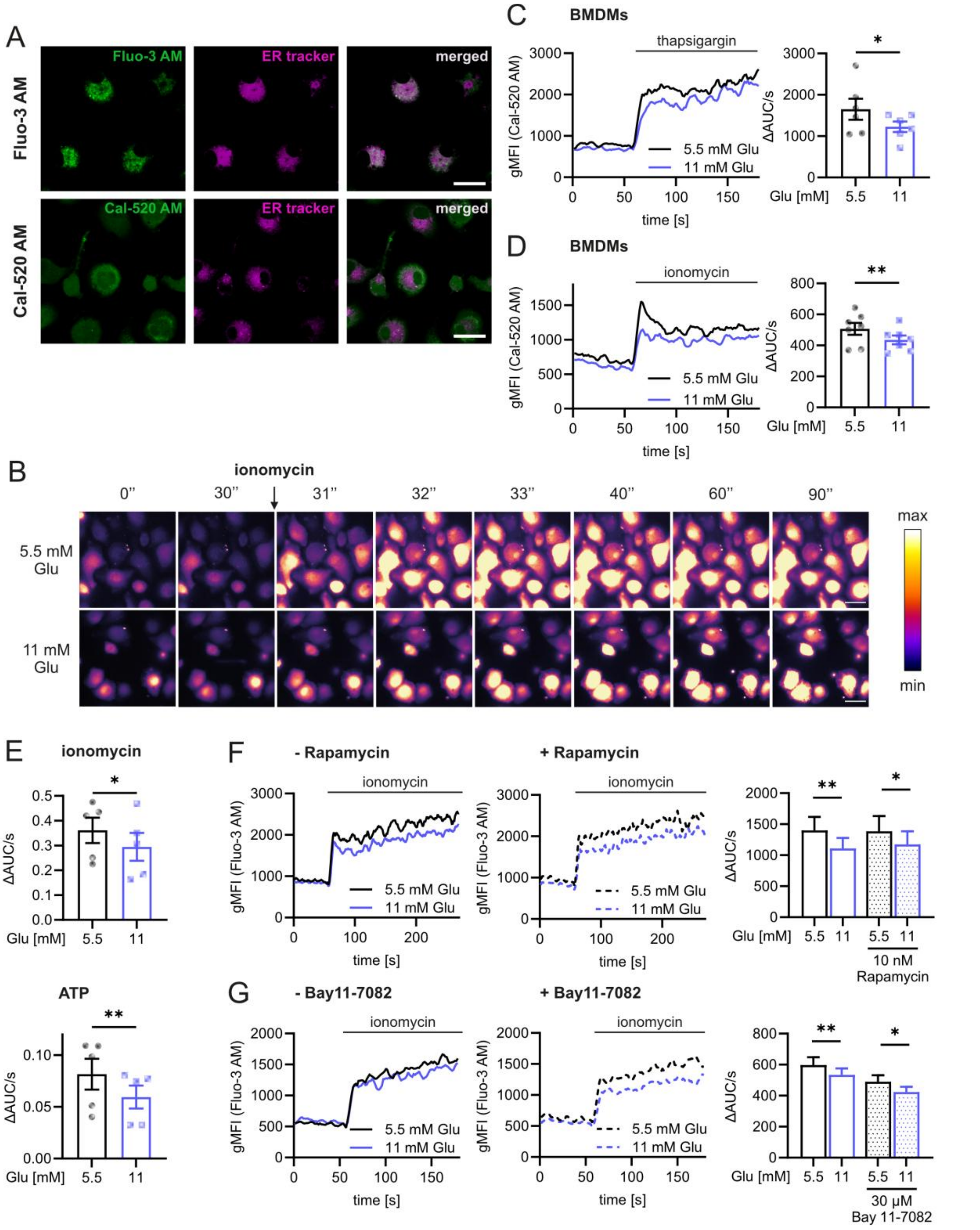

Figure S2, related to Figure 2. Glucose impairs Ca^{2+} signaling independent of mTOR and NF-κB

A) Immuno fluorescence microscopy of BMDMs treated with Cal-520 AM or Fluo-3 AM. The ER was stained using ER tracker Blue White DPX. Scale bar: 20 µm. B) BMDMs were treated with indicated concentrations of glucose for 48h and stained with Cal-520 AM. Cellular Ca^{2+} response to ionomycin (1 µg/mL) was monitored by live cell microscopy. Representative images from one out of eight

independent experiments. Scale bar: 30 µm. C) BMDMs were treated with indicated glucose concentrations for 48h and stained with Cal-520 AM. Changes in cytosolic Ca^{2+} concentrations were analyzed after addition of thapsigargin (1 µM) by flow cytometry. D) BMDMs were treated with indicated glucose concentrations for 48h and stained with Cal-520 AM. Changes in cytosolic Ca^{2+} concentrations were analyzed after addition of ionomycin (1 µg/mL) in the absence of extracellular Ca^{2+} by flow cytometry. E) BMDMs were treated with indicated glucose concentrations for 48h and stained with the ratiometric Ca^{2+} sensor FuraRed AM. Changes in cytosolic Ca^{2+} concentrations were analyzed after addition of ionomycin (1 µg/mL) or ATP (100 µM) by flow cytometry. F, G) BMDMs were treated with indicated concentrations of glucose in the presence of 10 nM rapamycin (F, 24h) or 30 µM Bay 11-7082 (G, 48h) and stained with Fluo-3 AM. Cellular Ca^{2+} response to ionomycin (1 µg/mL) was monitored.

Bar graphs are depicted as mean ± SEM. Ca^{2+} signaling curves are represented as pooled data from the same set of independent experiments. $^{**}p < 0.01$, $^{*}p < 0.05$; by paired Student's t test (C, D, E) or One-Way ANOVA corrected for multiple comparisons by the Tukey method (F, G). ΔAUC/s: difference of the Area under the Curve per second between after and before stimulation gMFI: geometric Mean Fluorescence Intensity; Glu: glucose; norm: normalized.

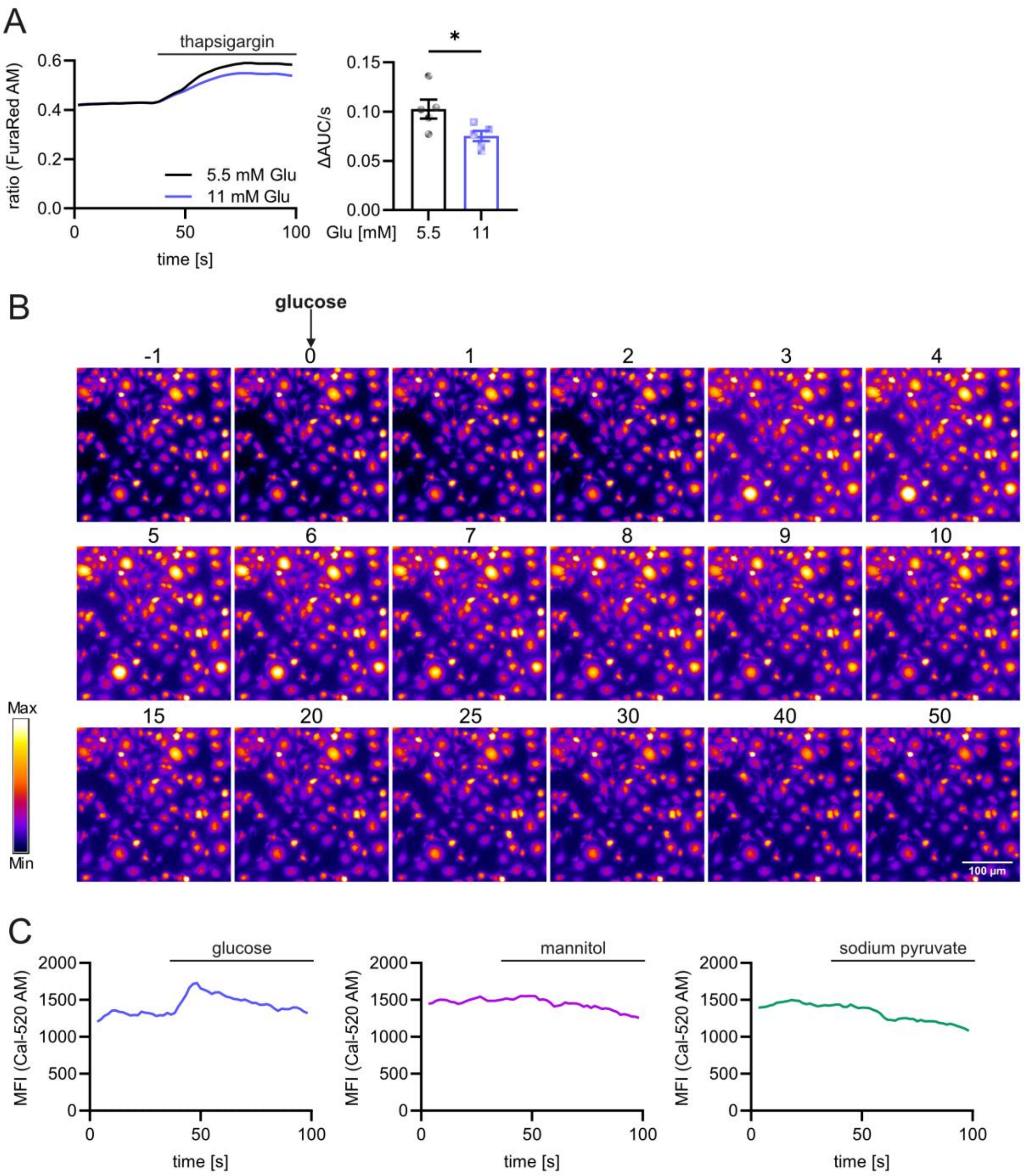

Figure S3, related to Figure 3. Glucose but not pyruvate induces intracellular Ca^{2+} signaling

A) BMDMs were treated with increased glucose concentrations for 48h and stained with the ratiometric Ca^{2+} indicator FuraRed. Ca^{2+} signals in response to thapsigargin (1 µM) were monitored in the absence of extracellular Ca^{2+} by flow cytometry. B) BMDMs were stained with Cal-520 AM. Changes in fluorescence intensity in response to addition of 22 mM glucose were monitored by live cell imaging. C) BMDMs were stained with Cal-520 AM. Changes in cytosolic Ca^{2+} concentrations were analyzed after addition of glucose, mannitol or sodium pyruvate (22 mM each) by flow cytometry. Ca^{2+} signaling curves are represented as pooled data from 5 independent experiments.

Bar graphs are depicted as mean ± SEM. Ca^{2+} release curves are represented as pooled data from the same set of independent experiments. $^{*}p < 0.05$ by paired Student's t test. ΔAUC/s: difference of the Area under the Curve per second between after and before stimulation; MFI: Mean Fluorescence Intensity; Glu: glucose.

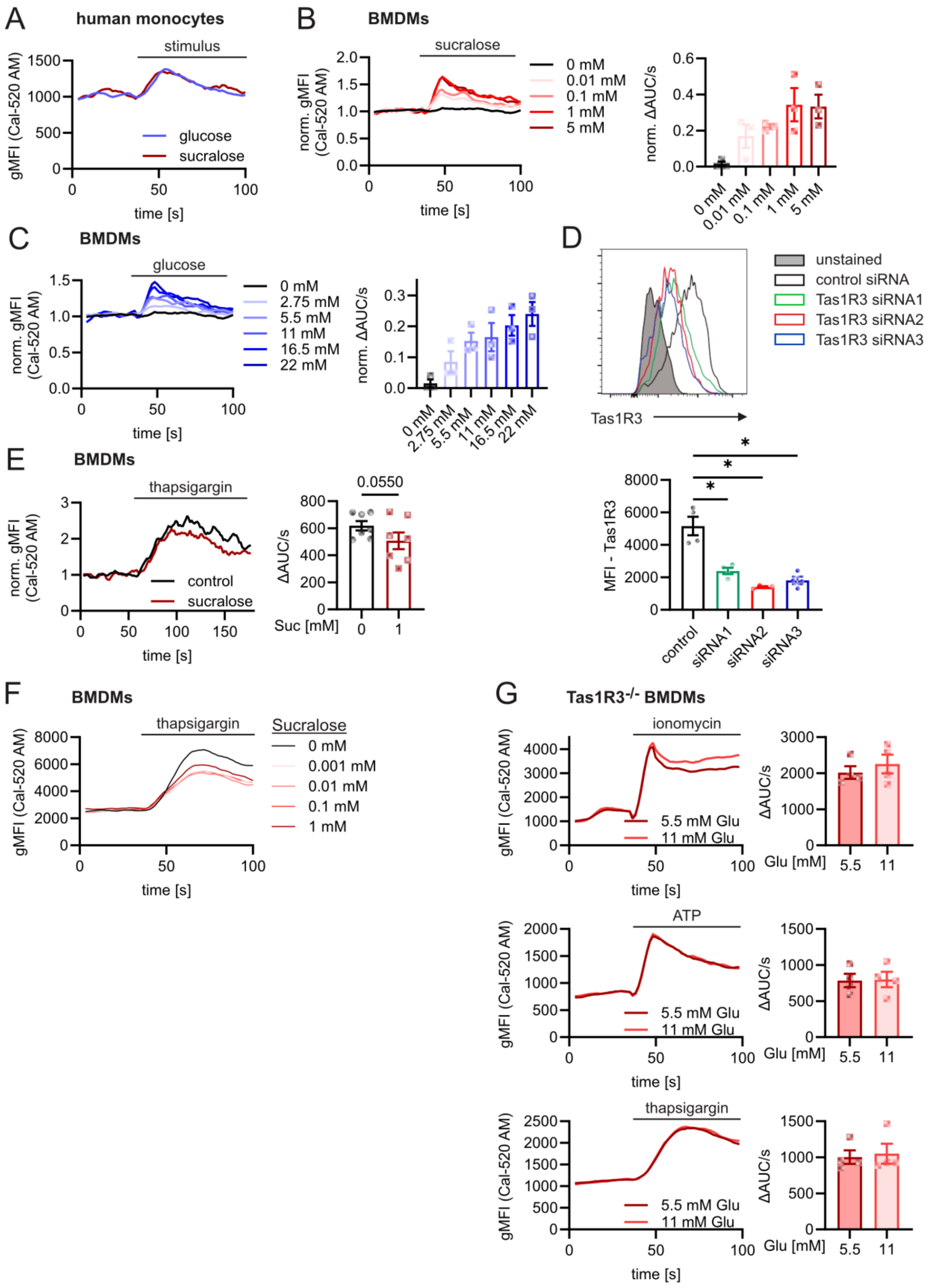

Figure S4, related to Figure 4. Glucose impairs cellular Ca^{2+} homeostasis by activation of the taste receptor Tas1R3

A) Human CD14$^+$ monocytes were stained with Cal-520 AM. Changes in cytosolic Ca^{2+} concentrations were induced by addition of 22 mM glucose or 5 mM sucralose and monitored by flow cytometry (n=4). B, C) BMDMs were stained with Cal-520 AM and cytosolic Ca^{2+} levels were monitored by flow cytometry. Cells were stimulated with different concentrations of sucralose (B) or glucose (C). D) Tas1R3 expression in BMDMs after down-regulation via siRNA. E, F) Changes in cytosolic Ca^{2+} concentrations in BMDMs, pretreated with indicated concentrations of sucralose (48h) and stained with Cal-520 AM, after stimulation with 1 µM thapsigargin, analyzed by flow cytometry. G) Ca^{2+} release into the cytosol in Tas1R3$^{-/-}$ BMDMs pretreated with the indicated glucose concentrations (48h) after stimulation with 1 µg/mL ionomycin, 100 µM ATP or 1 µM thapsigargin.

Bar graphs are depicted as mean ± SEM. Ca^{2+} signaling curves are represented as pooled data from the same set of independent experiments. $^*p < 0.05$ by One-Way ANOVA corrected for multiple comparisons by the Dunnett method (B, C, D, F) or paired Student's t test (E). ΔAUC/s: difference of the Area under the Curve per second between after and before stimulation; gMFI: geometric Mean Fluorescence Intensity; norm.: normalized.

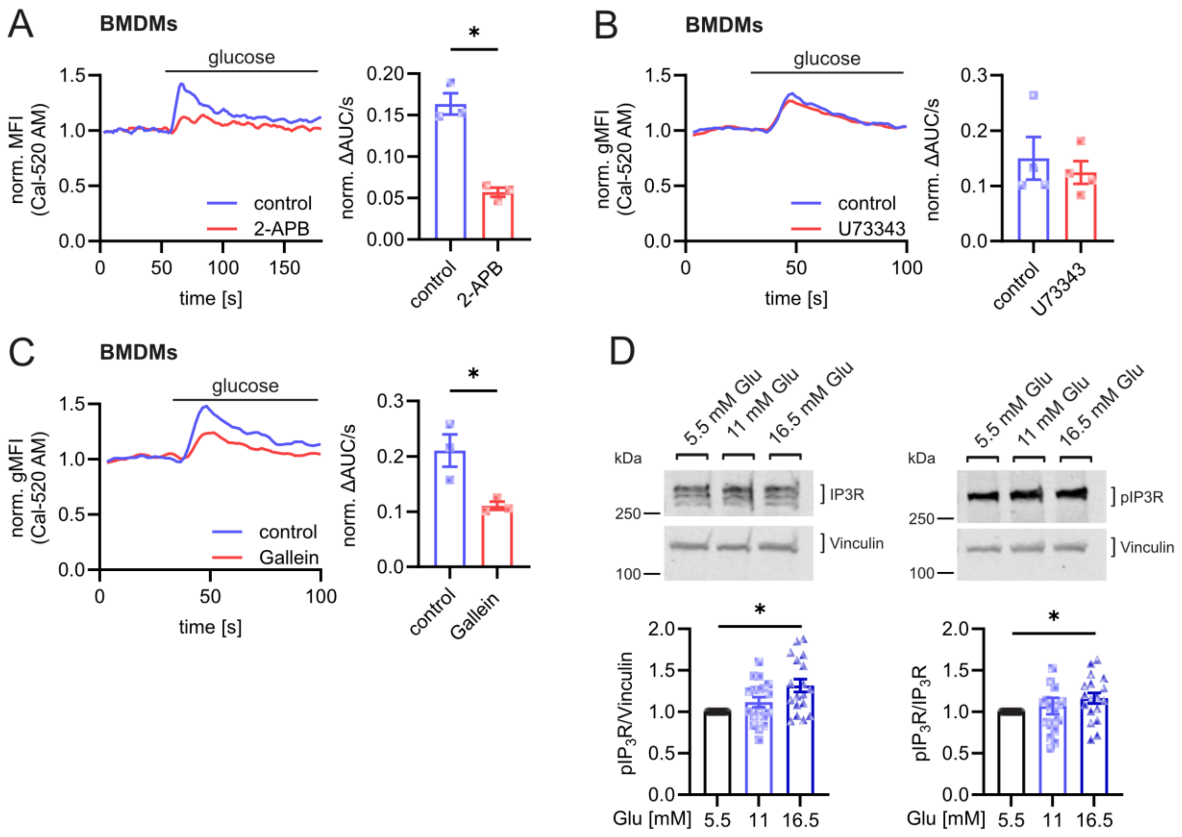

Figure S5, related to Figure 4. Glucose impairs cellular Ca^{2+} homeostasis by stimulation of the IP3R

A, B, C) BMDMs were stained with Cal-520 AM and incubated with 100 µM 2-APB (A), 5 µM U73343 (B) or 20 µM Gallein (C) for 10 min. Changes in cytosolic Ca^{2+} concentrations were monitored by flow cytometry after addition of 22 mM glucose. D) Expression and phosphorylation of IP3R in lysates from BMDMs treated with indicated glucose concentrations for 48h by western blot.

Bar graphs are depicted as mean ± SEM. Ca^{2+} signaling curves are represented as pooled data from the same set of independent experiments. $^{*}p < 0.05$ by paired Student's t test (A, C) or calculation of confidence intervals (D). ΔAUC/s: difference of the Area under the Curve per second between after and before stimulation; gMFI: geometric Mean Fluorescence Intensity; norm.: normalized.

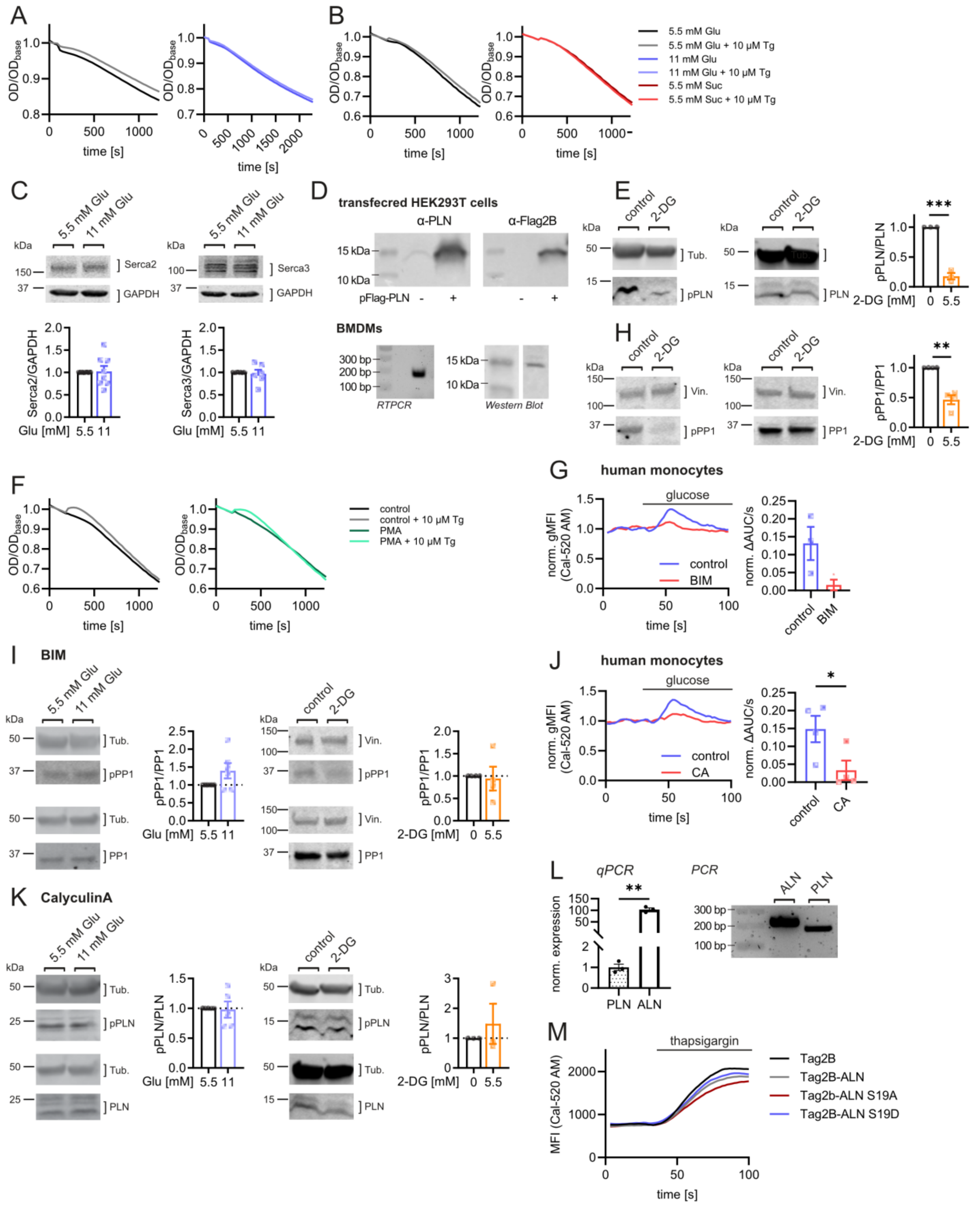

Figure S6, related to Figure 5. Taste receptor activation impairs SERCA activity via dephosphorylation of PP1 and PLN

A, B) SERCA activity in BMDMs after 48h of treatment with glucose (A) or sucralose (B). OD was normalized to baseline before addition of ATP. C) Expression of SERCA2 and SERCA3 by western blot. D) Amplified cDNA was cloned into pCMV-Tag2B, sequenced and expressed as FLAG fusion protein in HEK293T cells for western blot analysis using a PLN- or Flag-specific antibody (top).

Western blot and RT-PCR of PLN in BMDMs (bottom). E) Expression and phosphorylation of PLN after addition of 5.5 mM 2-DG for 4h by western blot. F) SERCA activity in BMDMs after treatment with 100 nM PMA for 24h. OD was normalized to baseline before addition of ATP. G) Human $CD14^{+}$ monocytes were stained with Cal-520 AM, incubated with 5 µM BIM for 10 min. Changes in cytosolic Ca^{2+} concentrations were monitored by flow cytometry after addition of 22 mM glucose. H) Expression and phosphorylation of PP1 after addition of 5.5 mM 2-DG for 4h by western blot. I) Expression and phosphorylation of PP1 after addition of 5.5 mM 2-DG or elevated glucose concentrations and 5 µM BIM for 4h. J) Human $CD14^{+}$ monocytes were stained with Cal-520 AM, incubated with 100 nM CA for 10 min. Ca^{2+} release into the cytosol was monitored by flow cytometry after addition of 22 mM glucose. K) Expression and phosphorylation of PLN after addition of 5.5 mM 2-DG or elevated glucose concentrations and 100 nM CA for 4h. L) Expression of ALN and PLN in BMDMs monitored via qPCR and PCR. M) HEK293T cells were transfected with ALN, ALN S19A and ALN S19D and stained with Cal-520 AM. Changes in cytosolic Ca^{2+} concentrations were monitored by flow cytometry after addition of 1 µM thapsigargin.

Bar graphs are depicted as mean ± SEM. Ca^{2+} signaling curves are represented as pooled data from the same set of independent experiments. $^{***}p < 0.001$, $^{**}p < 0.01$, $^{*}p < 0.05$ by paired Student's t test (G, J, L) or calculation of confidence intervals (C, E, H, I, K). ΔAUC/s: difference of the Area under the Curve per second between after and before stimulation; ALN: another-regulin; BIM: bisindolylmaleimide; CA: Calyculin A; gMFI: geometric Mean Fluorescence Intensity; Glu: glucose; norm.: normalized; OD: optical density; PLN: phospholamban; PP1: protein phosphatase 1; Tg: Thapsigargin; Tub: tubulin; Vin: vinculin.

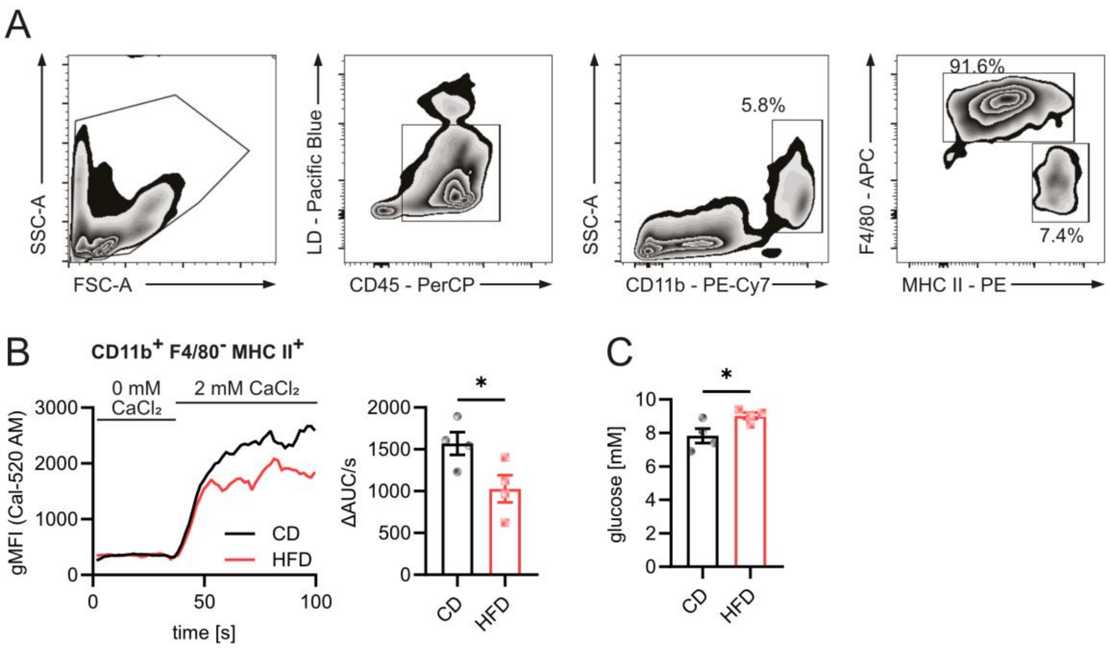

Figure S7, related to Figure 6. Flow cytometric analysis and reduced Ca^{2+} response in HFD-treated peritoneal macrophages

A) Gating strategy to discriminate small (MHC II^{+}) and large ($F4/80^{+}$) peritoneal macrophages ($CD11b^{+}$). Cells from peritoneal lavage were stained with the Ca^{2+}-sensitive dye Cal-520 AM, viability dye (LD) and antibodies against CD45, CD11b, F4/80, MHC II. B) Peritoneal macrophages from HFD- or CD-fed mice were stained with Cal-520 AM. Changes in cytosolic Ca^{2+} concentrations after addition of 2 mM extracellular $CaCl_2$ were analyzed by flow cytometry. C) Blood glucose concentrations of mice shown in (B).

Bar graphs are depicted as mean ± SEM. Ca^{2+} signaling curves are represented as pooled data from the same set of independent experiments. $*p < 0.05$ by paired Student's t test. ΔAUC/s: difference of the Area under the Curve per second between after and before stimulation; gMFI: geometric Mean Fluorescence Intensity; HFD: high fat diet; CD: control diet.

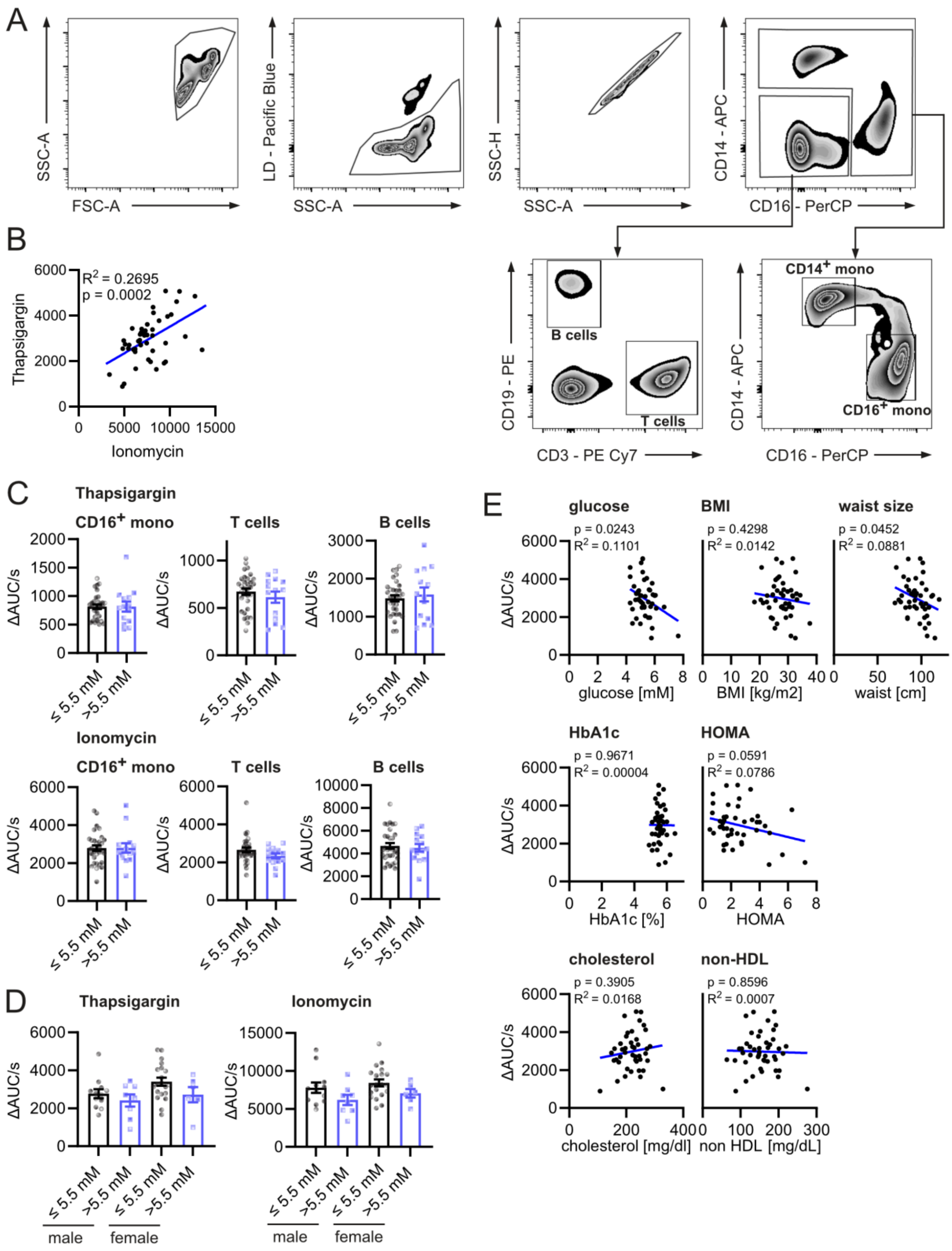

Figure S8, related to Figure 6. Negative correlation of Ca^{2+} signaling and fasting blood glucose concentrations in a human cohort

A) Gating strategy for different PBMC populations. Cells were stained with the Ca^{2+}-sensitive dye Cal-520 AM, viability dye (LD) and antibodies against CD3, CD14, CD16 and CD19. B) Correlation of Ca^{2+} response induced by ionomycin (1 µg/mL) and thapsigargin (1 µM) in CD14$^+$ monocytes from

a human cohort containing 32 healthy (fasting blood glucose ≤ 5.5 mM) and 14 prediabetic (fasting blood glucose > 5.5 mM) donors. C) Changes in cytosolic Ca^{2+} concentrations induced by 1 µM thapsigargin (top) or 1 µg/mL ionomycin (bottom) in $CD16^{+}$ non-classical monocytes, T cells and B cells from the human cohort. D) Changes in cytosolic Ca^{2+} concentrations induced by 1 µM thapsigargin (left) and 1 µg/mL ionomycin (right) separated by donor gender. E) Linear regression analysis of Ca^{2+} release intensity induced by thapsigargin (1 µM) in $CD14^{+}$ monocytes and blood glucose, BMI, waist size, HbA1c, HOMA index, cholesterol and non-HDL cholesterol.

Bar graphs are presented as mean ± SEM. ΔAUC/s: difference of the Area under the Curve per second between after and before stimulation.

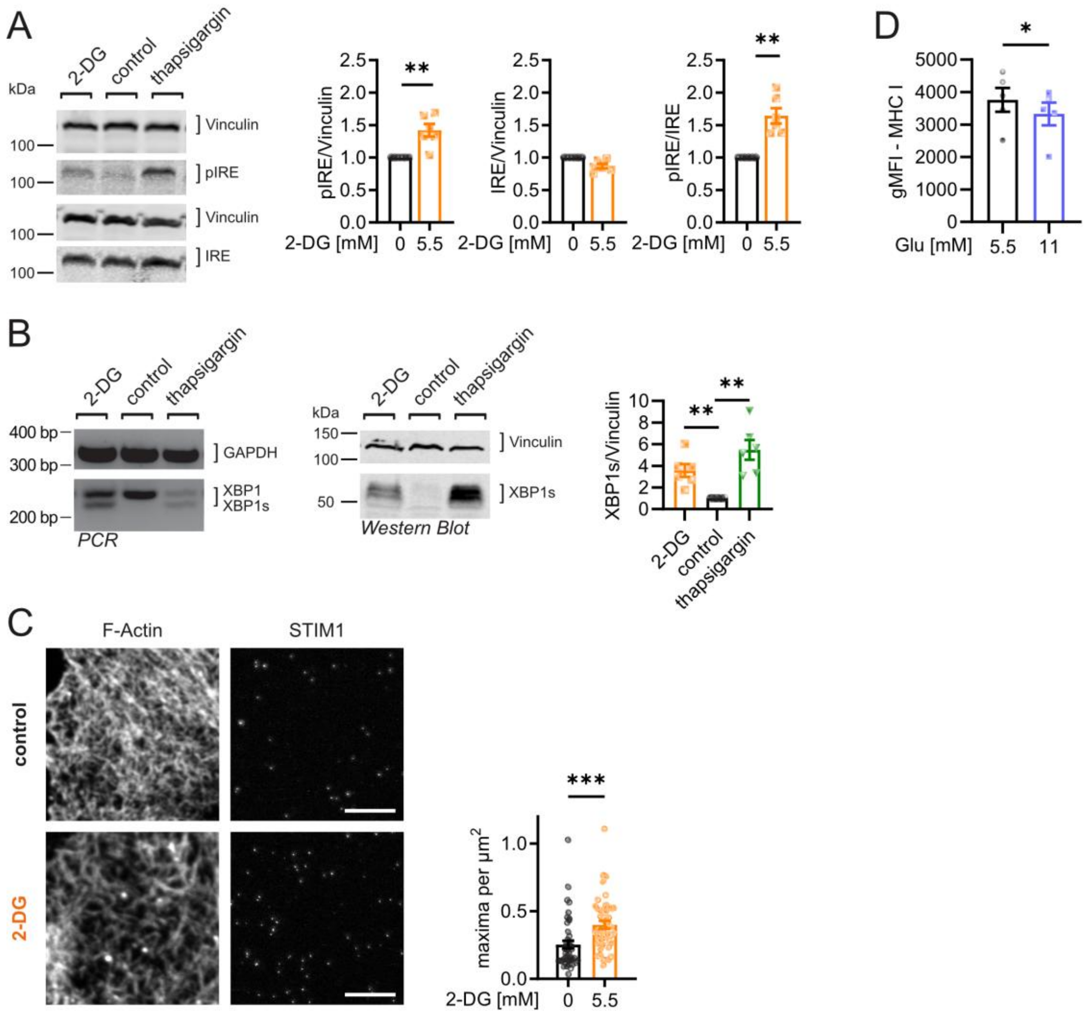

Figure S9, related to Figure 7. Glucose and 2-DG mediate ER

A) Phosphorylation of IRE1α after treatment with 5.5 mM 2-DG for 4h monitored by western blot. B) Splicing of XBP1 in BMDMs treated with 2-DG (5.5 mM) or thapsigargin (500 nM) for 4h monitored by western blot and RT-PCR. C) BMDMs were treated with 5.5 mM 2-DG for 3h and membrane sheets were generated by sonication and stained with Phalloidin (left) or STIM (right) and analyzed by confocal (Phalloidin) or STED microscopy (STIM). Scale bar: 3 µm. D) MHC I staining of BMDMs treated with the indicated concentrations of glucose for 48h.

Data are presented as mean ± SEM, $^{*}p < 0.05$; $^{**}p < 0.01$; $^{***}p < 0.001$ by calculation of confidence intervals (A), paired Student's t test (C, D), one-way ANOVA corrected for multiple comparisons by the Tukey method (B). Glu: glucose; 2-DG: 2-deoxy-glucose.

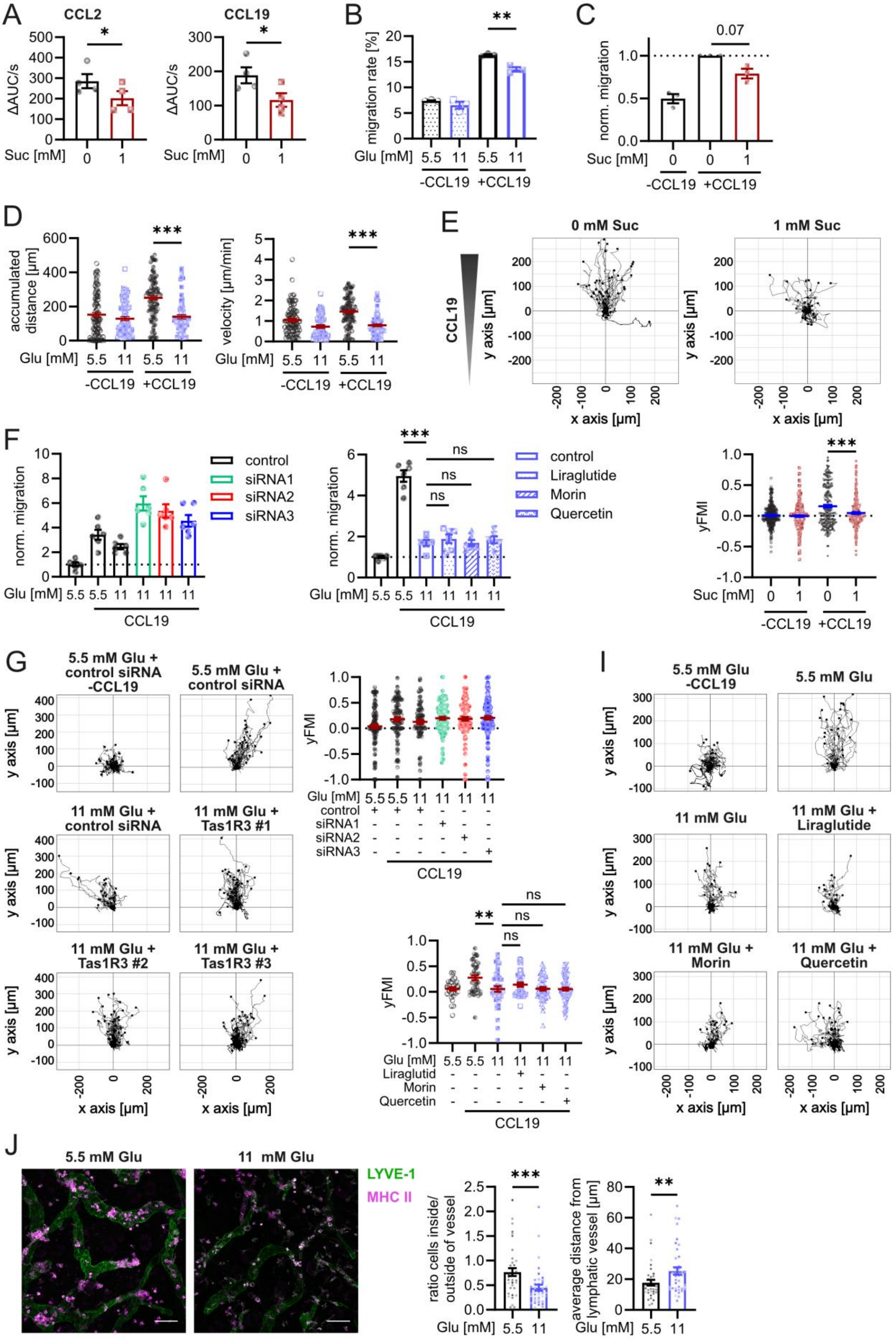
A
CCL2
CCL19
ΔAUC/s
Suc [mM]
B
migration rate [%]
Glu [mM]
-CCL19
+CCL19
C
0.07
norm. migration
D
accumulated distance [µm]
velocity [µm/min]
E
0 mM Suc
1 mM Suc
CCL19
y axis [µm]
x axis [µm]
F
control
siRNA1
siRNA2
siRNA3
Liraglutide
Morin
Quercetin
ns
yFMI
G
5.5 mM Glu + control siRNA -CCL19
5.5 mM Glu + control siRNA
11 mM Glu + control siRNA
11 mM Glu + Tas1R3 #1
11 mM Glu + Tas1R3 #2
11 mM Glu + Tas1R3 #3
Liraglutid
I
5.5 mM Glu -CCL19
5.5 mM Glu
11 mM Glu
11 mM Glu + Liraglutide
11 mM Glu + Morin
11 mM Glu + Quercetin
J
5.5 mM Glu
11 mM Glu
LYVE-1
MHC II
ratio cells inside/ outside of vessel
average distance from lymphatic vessel [µm]

Figure S10, related to Figure 7. Glucose and sucralose reduce macrophage migration

A) Intensities of changes in cytosolic Ca^{2+} concentrations in response to 14.5 nM CCL2 or 500 ng/mL CCL19 after treatment with 1 mM sucralose for 48h. B, C) Transwell migration assay of LPS-induced BMDMs treated with elevated glucose (B) or sucralose (C) concentration (48h) towards CCL19 (200 ng/mL). D, E) BMDMs were treated with indicated glucose (D) or sucralose (E) concentrations and stimulated with LPS. Migration towards CCL19 (1000 ng/mL) was analyzed using 3D collagen type I gels. Accumulated distance and velocity were analyzed from time series of live cell imaging. Quantifications depict pooled data from 3 independent experiments with at least 70 cells per condition and region of interest. Migration tracks are shown from one exemplary experiment. F, G) Transwell (F) or 3D collagen type I gel migration assay (G) of LPS-induced BMDMs treated with elevated glucose (11 mM) after downregulation of Tas1R3. H, I) Transwell (H) or 3D collagen type I gel migration assay (I) of LPS-induced BMDMs treated with glucose and ER stress inhibitors liraglutide (100 nM), morin (25 μM) or quercetin (25 μM). J) *In situ* migration in explanted ear sheets incubated with increased glucose concentrations for 48h. Lymphatic vessels were stained for Lyve-1, migratory cells for MHC II. Data show exemplary Z projections (left) and calculated distance of cells to the lymphatic vessels and ratio of cells in and outside of vessels (right). Each data point represents one field of view pooled from ear sheets from 4 different mice. Scale bar: 100 μm.

Data are presented as mean ± SEM, $^{*}p < 0.05$; $^{**}p < 0.01$; $^{***}p < 0.001$ by calculation of paired Student's t test (A), one-way ANOVA corrected for multiple comparisons by the Tukey method (B, C, D, F, H) and Mann-Whitney test (J). ΔAUC/s: difference of the Area under the Curve per second between after and before stimulation; gMFI: geometric Mean Fluorescence Intensity; Glu: glucose; 2-DG: 2-deoxy-glucose; ns: not significant; Suc: Sucralose.

Mean ± SEM	**≤ 5.5 mM**	**> 5.5 mM**
**Proband**	N = 32	N = 14
**Sex**	F = 20, M = 12	F = 6, M = 8
**Age**	63.65 ± 0.61	65.86 ± 1.44
**BMI [kg/m^2]**	27.37 ± 0.92	28.91 ± 0.76
**Glucose [mM]**	4.96 ± 0.08	6.02 ± 0.14***
**HbA1c [%]**	5.46 ± 0.03	5.82 ± 0.08***
**Waist [cm]**	92.48 ± 2.43	98.96 ± 2.59
**Non-HDL cholesterol [mg/dL]**	153.75 ± 5.77	154.64 ± 13.41
**Triglyceride [mg/dL]**	116.06 ± 7.44	140.86 ± 13.8
**HOMA**	2.06 ± 0.14	3.5 ± 0.50*
**Cholesterol [mg/dL]**	221.84 ± 5.49	211.43 ± 13.76

Table S1, related to Figure 6. Metadata and clinical parameters from human cohort

*$p < 0.05$; ***$p < 0.001$ by calculation of unpaired Student’s t test
